# The landscape of DNA repeat elements in human heart failure

**DOI:** 10.1186/gb-2012-13-10-r90

**Published:** 2012-10-03

**Authors:** Syed Haider, Lina Cordeddu, Emma Robinson, Mehregan Movassagh, Lee Siggens, Ana Vujic, Mun-Kit Choy, Martin Goddard, Pietro Lio, Roger Foo

**Affiliations:** 1 Computer Laboratory, William Gates Building, University of Cambridge, JJ Thomson Avenue, Cambridge CB3 0FD; 2 Division of Cardiovascular Medicine, University of Cambridge, Addenbrooke's Centre for Clinical Investigation, Level 6, Hills Road, Cambridge, CB2 0QQ; 3 Department of Histopathology, Papworth Hospital, Papworth Everard, Cambridge, UK; 4 Cardiovascular Research Institute, National University Health System, Singapore; 5 Genome Institute of Singapore, Singapore 138672, Singapore

## Abstract

**Background:**

The epigenomes of healthy and diseased human hearts were recently examined by genome-wide DNA methylation profiling. Repetitive elements, heavily methylated in post-natal tissue, have variable methylation profiles in cancer but methylation of repetitive elements in the heart has never been examined.

**Results:**

We analyzed repetitive elements from all repeat families in human myocardial samples, and found that satellite repeat elements were significantly hypomethylated in end-stage cardiomyopathic hearts relative to healthy normal controls. Satellite repeat elements are almost always centromeric or juxtacentromeric, and their overexpression correlates with disease aggressiveness in cancer. Similarly, we found that hypomethylation of satellite repeat elements correlated with up to 27-fold upregulation of the corresponding transcripts in end-stage cardiomyopathic hearts. No other repeat family exhibited differential methylation between healthy and cardiomyopathic hearts, with the exception of the Alu element SINE1/7SL, for which a modestly consistent trend of increased methylation was observed.

**Conclusions:**

Satellite repeat element transcripts, a form of non-coding RNA, have putative functions in maintaining genomic stability and chromosomal integrity. Further studies will be needed to establish the functional significance of these non-coding RNAs in the context of heart failure.

## Background

One of the greatest surprises of high-throughput transcriptome analysis in recent years has been the discovery that the mammalian genome is pervasively transcribed into many different complex families of RNA [1]. Up to 40% of the transcriptome has no protein coding capacity and different forms of non-coding RNA include piwi-interacting RNA (piRNA), small nucleolar RNA (snoRNA), long non-coding RNA and others [2]. Of these, microRNA is the only non-coding RNA that is currently the most well-studied in cardiovascular research. Repetitive elements and retrotransposons make up at least 45% of the human genome and are expressed as non-coding transcripts in different tissues [3,4] but their expression in the heart has never been examined. In the adult brain, long interspersed nuclear element-1 (LINE-1) retrotransposons were unexpectedly discovered to undergo transcription, active mobilization and large-scale insertion and copy-number expansion [5]. LINE-1 expansion was ascribed to neuroprogenitor cells in adult brains and LINE-1 retrotransposition may explain genetic diversity and differential neuronal properties between the brains of different individuals, and also the different types of neurons in the brain of an individual [5].

The term 'repetitive element' refers to DNA sequences that are present in multiple copies in the genomes in which they reside. Repetitive elements are subdivided into (i) interspersed sequences (LINEs and SINEs) derived from non-autonomous or autonomous transposable elements, and (ii) tandem array repeats of simple or complex sequences (satellite elements). Interspersed LINEs and SINEs are found throughout the genome, whereas satellite (SAT) elements are largely confined to centromeres or centromere-adjacent (juxtacentromeric) heterochromatin. Satellite-α (SATα) repeats are composed of 170 bp DNA sequences and represent the main DNA component of every human centromere [6]. Satellite 2 (Sat2) repeats are found in juxtacentromeric heterochromatin and are most abundant in the long juxtacentromeric region of chromosome 1. A collective feature of repetitive elements genome-wide is that of DNA methylation. DNA methylation refers to the epigenetic modification in which the cytosine nucleotide is modified by a methyl-group in the carbon-5 position. DNA sequences of repetitive elements are highly methylated in postnatal tissues but can be variably methylated in cancer [7]. Methylation of repetitive elements contributes to the heterochromatic structure of their genomic loci and explains why they are transcriptionally silent. In a genome-wide DNA methylation screen of nerve sheath tumors, Beck and colleagues [8] found that SAT repeats, but not other repetitive elements, are hypomethylated and aberrant methylation of these was associated with the transition from healthy cells to malignant disease.

Little is known about DNA repetitive elements in the cardiac genome. In 1990 Gaubatz and Cutler [9] reported that SAT repeats are actively transcribed in hearts of old mice (aged 12 to 32 months) compared to young (2 to 6 months). This was in contrast to the absence of any difference in transcripts of SINEs and LINEs. Repetitive elements from diseased hearts were, however, not examined.

Our group recently reported the first genome-wide differential DNA methylation study in end-stage cardiomyopathic (EsCM) human hearts and gave a glimpse of the distinct patterns of DNA methylation profiles in EsCM compared to healthy age-matched controls (CTRL) [10]. We found significant differential methylation in the tandem repeat array at the subtelomeric *DUX4 *locus that associated with differential *DUX4 *expression. This prompted us to extend our analysis to the genome-wide methylation profile of all other repetitive elements in the cardiac genome.

## Results

### DNA methylation mapping of human repeat sequences

**Figure 1:**
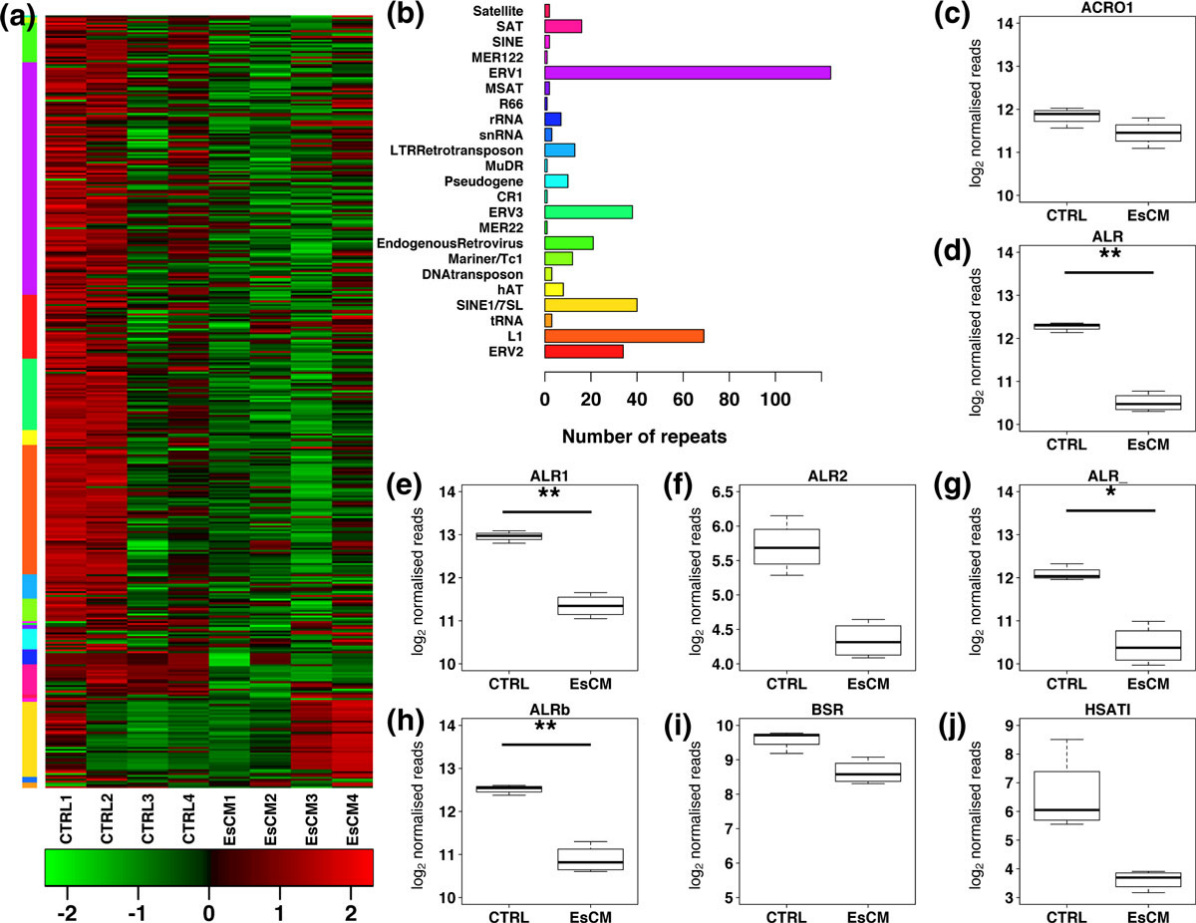
**Summary of count data and candidate SAT repeat elements**. **(a) **Heatmap of log2 normalized read counts for CTRL and EsCM patients across repeat elements. The rows were scaled such that every row has μ = 0 and σ = 1. The color bar on the vertical axis represents families of repeat elements. A fully annotated large-scale heatmap is available in Additional file 3. **(b) **A bar chart representing the number of repeat sequences per family. **(c-j) **The groupwise log2 normalized read counts. The two groups were compared using unpaired Welch's *t*-test followed by adjustment for multiple comparisons. *ALR_ *methylation was significantly different between the CTRL and EsCM group (* *P *< 0.05) while *ALR*, *ALR1*, and *ALRb *methylation levels were highly significantly different between the two groups (** *P *< 0.01).

To systematically evaluate differential methylation of repetitive elements in the cardiac genome, we took a two-step approach depicted in Additional file 1. First, the methylated DNA immunoprecipitation (MeDIP)-seq dataset from four EsCM hearts and four normal left ventricular (LV) tissue samples (CTRL) (Additional file 2) published previously was re-examined specifically for DNA repetitive element methylation. Corresponding to the hypothesis that there is a convergent 'unifying pathway' of gene expression that characterizes end-stage failing hearts regardless of the original inciting cause, and that this reflects other 'unifying pathway' processes such as fibrosis, angiogenesis and cell death in end-stage heart failure [11], DNA methylation profiles in our previous analyses did not differ between ischemic and idiopathic cardiomyopathic hearts [10,12]. We therefore used all ischemic and idiopathic cardiomyopathic samples as collectively representative of EsCM [10,12]. High-throughput sequencing from MeDIP had generated a total of approximately 127 million reads [10]. Reads were mapped to the human reference genome assembly Hg18 and to repeat sequences in Repbase [13,14]. Uniquely mapped reads were normalized and subsequently compared between CTRL and EsCM for all repetitive elements of the genome (Figure 1a,b; Additional files 2, 3 and 4). Since the sample size was small, we opted for one to one comparison between the two groups using Fisher's exact test. This resulted in 16 pairwise comparisons between CTRL and EsCM samples (Additional files 5 and 6). In order to identify differentially methylated repetitive elements (DMReps), we used a simple guide to keep those repetitive elements whereby Fisher's exact test statistic was significant (*P *< 0.05) for at least 14 out of the total 16 pairwise comparisons. For the purpose of our comparison, we observed that SINE-1 (SINE1/7SL), LINE-1 (L1), Satellite (SAT) and endogenous retrovirus 1 (ERV1) families were highly representative, having 32, 13, 8 and 8 repeat sequences, respectively. The three families SINE1/7SL, L1, and ERV1 featured a mixed trend of hypo- and hypermethylation between EsCM and CTRL (Additional files 5 and 6). Therefore, it was not possible to derive any conclusions with these three families from our dataset. In marked contrast, the identified DMReps were significantly enriched for SAT repeats (*P *= 4.12 × 10^-3^, hypergeometric test; *P *= 4.10 × 10^-3^, permutation analysis) (Figure 1c-j). Moreover, the SAT family demonstrated a consistent trend of hypomethylation in EsCM across our comparisons (green only in Additional files 5 and 6). As a complementary approach, the two groups were also compared using unpaired Welch's *t*-test, identifying five DMReps (*P*-adjusted <0.05; Figure 1d,e,g,h; Additional file 7). Unsurprisingly, the results were similar to the pairwise comparison as four out of five of these DMReps were also significantly enriched for SAT repeats (*ALR*, *ALR_*, *ALRb *and *ALR1*; *P *= 7.51 × 10^-6^, hypergeometric test; *P *= 5.0 × 10^-6^, permutation analysis).

To assess the classification of identified DMReps in more detail, we grouped repeat sequences into respective families (Additional file 8) and classes (Additional file 9) using Repbase annotations. As expected, the overall landscape of repeat families was a cumulative representative of its member repeats. The Fisher's exact test statistic was highly significant (*P *< 0.01) across all 16 SAT family comparisons between EsCM and CTRL samples. SAT hypomethylation in EsCM samples was also found when the comparisons were made between repeat classes (Fisher's *P *< 0.01). Family-wise and class-wise comparisons did not demonstrate the same consistency of either hypo- or hypermethylation for any other group of repeat sequences.

We therefore chose to limit our subsequent analysis to the four SAT repeats identified by both methods as described above. However, analysis for *ALR1 *had to be excluded because, technically, we found that we could not design any primer pairs that were specific only for *ALR1*. Our analysis therefore focused on *ALR*, *ALR_ *and *ALRb*. Global coordinates for each of these remaining three SAT repeats were carefully annotated (Additional file 10). We proceeded to validate our finding of SAT hypomethylation in EsCM patients by analyzing the methylation density averaged for each of the three global sets of coordinates, including their flanking genomic locations, using the previously established BATMAN algorithm [10]. All three SAT repeats showed a reduction in methylation density in EsCM samples (Additional file 11), consistent with the analysis in Figure 1 and Additional files 5 and 6.

The lack of SAT element enrichment detected in EsCM by MeDIP may be explained by an artifact of copy-number contraction of SAT elements within the genome of EsCM compared to CTRL, and not necessarily an enrichment because of relative hypomethylation. We therefore quantified SAT copy-number by quantitative PCR (qPCR) of genomic DNA from all our LV samples. Contrary to copy-number contraction in EsCM, a trend for more SAT elements was found in EsCM (Additional file 12). This confirmed that differential enrichment of SAT elements by MeDIP reflected differential methylation and not differential genomic SAT copy number.

### Hypomethylation of SAT repetitive elements correlated with increased SAT transcription

**Figure 2:**
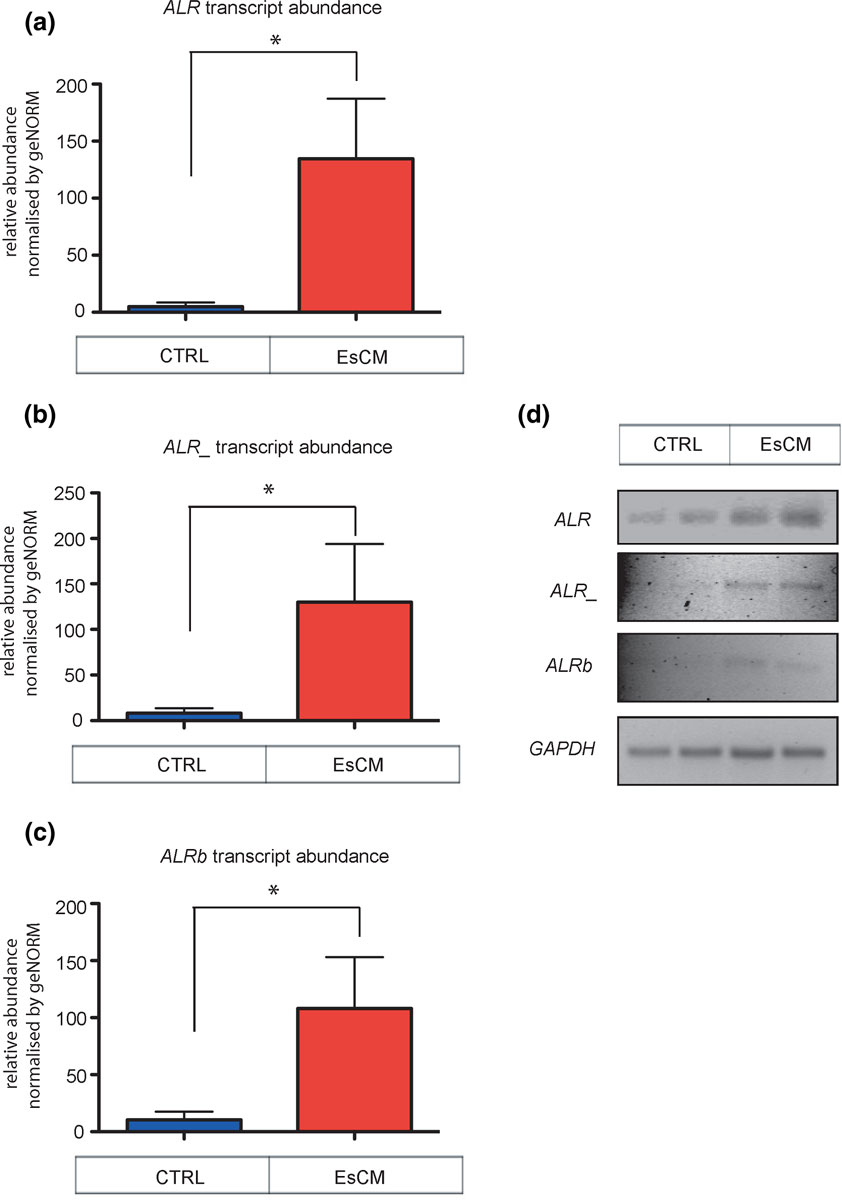
**Quantitative RT-PCR for the transcript abundance of SAT family repeat sequences (*ALR*, *ALR*_ and *ALRb*)**. **(a-c) **Quantification of transcript abundance for *ALR *(a), *ALR*_ (b) and *ALRb *(c) repeat elements was performed on a panel of EsCM and CTRL LV samples (EsCM A to H and CTRL 1 to 16, according to Additional file 13), and normalized by geNORM obtained from housekeeping transcripts *RPLPO *and *TBP*. The two groups were compared using unpaired Wilcoxon rank-sum test. All three repeat elements were found to be significantly different from their respective CTRL group. Values shown are mean ± s.e.m. * *P *< 0.05. **(d) **Products from the qPCR reaction were run in a 2% agarose gel as shown. PCR reactions were TOPO-cloned and sequenced (not shown).

We have previously demonstrated that hypomethylation of DNA regulatory elements and loci in the cardiac genome associated with differential gene expression at the corresponding locus [10,12]. Moreover others have reported increased transcription from major SAT repeats in aged murine hearts in relation to the progressive loss of silencing of heterochromatin around centromeres [9]. We therefore tested the RNA abundance of *ALR*, *ALR_ *and *ALRb *repeats in CTRL and EsCM (Additional file 13, CTRL A to H and EsCM 1 to 16) by RT-qPCR. Transcripts of all three SAT repeat elements were significantly upregulated in EsCM compared to CTRL by up to 27-fold (Figure 2a-c). We further ascertained that only a single product was amplified from each PCR (Figure 2d) and PCR products were TOPO-cloned and sequence validated (not shown).

The chromatin mark of H3K36me3 (tri-methylated histone H3 at lysine 36) demarcates actively transcribed genomic loci [15]. We therefore used our previously published dataset of H3K36me3 chromatin immunoprecipitation (ChIP)-seq [10] and validated that the read density for this histone mark was enriched in the global coordinates for each of *ALR*, *ALR_ *and *ALRb *in EsCM compared to CTRL (Additional file 14). This was again consistent with the finding of increased transcription activity at the loci of these three SAT family members.

### Proximal genes to SAT repetitive elements

In order to propose a functional role linking hypomethylation of SAT elements to protein coding genes, the genomic loci of these elements were scanned for genes in proximity. We extended our search to 5,000 bp up- and downstream of SAT repeats that overlapped any known genes. Remarkably, almost all SAT repetitive elements were centromeric or juxtacentromeric and therefore did not have any genes associated with them, except for *ANKRD30BL *and *TRIM48*. These had *ALRb *and *ALR_ *repetitive elements overlapping within 5,000 bp upstream (Additional file 15). *TRIM48 *is a protein-coding gene and *ANKRD30BL *is a putative non-coding RNA. RT-qPCR for transcript abundance of *TRIM48 *and *ANKRD30BL *did not show any differential expression between healthy CTRL and EsCM hearts (not shown).

### Hypermethylation of SINE1/7SL repeat elements across EsCM patients

The other family with methylation differences suggested by our analyses was SINE1/7SL. This SINE1 sequence comprises Alu retrotransposons and is believed to have arisen from the gene that encodes 7SL RNA [16,17]. Alu repeats are linked with various cancer types featuring hypomethylation of oncogenes and hypermethylation of tumor supressors [18-20]. We noticed that 32 SINE1/7SL repeat elements possessing sufficient coverage demonstrated a modestly consistent trend of hypermethylation across EsCM. Although the trend was not conclusive for the CTRL 1 sample, the rest of the comparisons between EsCM and CTRL samples indicated hypermethylation in EsCM (Additional files 5 and 6). The merged results of the count data of these repeat elements within SINE1/7SL also demonstrated a similar trend (Additional files 8 and 9). Altogether, these pointed to a trend toward increased Alu methylation in EsCM.

## Discussion

High-throughput sequencing of RNA provides an unprecedented opportunity to examine the pervasive transcription of the mammalian genome [1]. While RNA-seq studies performed in the context of heart failure have demonstrated a wide variety of protein and non-protein coding transcripts that are up- and down-regulated in the diseased myocardium [21,22], transcripts arising from DNA repetitive elements in the cardiac genome have yet to be highlighted. Historically, repetitive DNA sequences have been refractory to many experimental approaches, particularly array-based ones that are dependent on hybridization. This problem is circumvented by MeDIP-seq because high-throughput sequencing provides excellent coverage for all major repeats [8]. High-throughput sequencing may have other intrinsic disadvantages, such as GC-dependent differential amplification of sequences, but our work involving the comparison between healthy and diseased tissue means that such shortcomings apply equally to both sets of tissue. Hence, this emphasizes the significance of our finding of DMReps in SAT elements in EsCM heart. Our analysis also hints at Alu element hypermethylation in EsCM heart but the more compelling results with SAT DMReps convinced us to focus our study on SAT repetitive elements.

SAT repetitive elements are mainly centromeric or juxtacentromeric. Centromeres are marked by a distinct set of histone variants and organized into blocks of nucleosomes. Clear evidence shows that the specification and propagation of centromeres are not defined by the underlying DNA sequence but rather by epigenetic mechanisms such as the histone variants and, possibly, DNA methylation[23,24]. Methylation changes or changes in histone modifications at these repetitive elements may hence predispose to increased transcription of the underlying SAT elements. Our findings of increased SAT transcript expression correlating with SAT hypomethylation in EsCM heart indeed correspond to our previous report of hypermethylation of the *DUX4 *subtelomeric tandem repeat and downregulation of the *DUX4 *transcript [10].

Overexpression of centromeric SAT transcripts in diseased hearts is reminiscent of centromeric-derived transcript upregulation in the conditional gene-targeted knockout of Dicer in embryonic stem cells [25]. There, Dicer deficiency also generates defects in methylation of centromeric DNA and overexpression of SAT repeats. SAT repeats are transcribed into non-coding RNAs that are implicated in fundamental processes, including gene silencing and maintenance of chromosomal integrity [26]. Like other non-coding RNA, the role of SAT transcripts seems likely to depend upon RNA-protein complexes. SAT transcripts assemble nucleoproteins at the centromere by directly binding to core centromeric proteins [27]. A direct interaction between splicing factors and SAT transcripts also recruits splicing factors to nuclear stress bodies during conditions of cellular stress [28]. Up to 100-fold upregulation of juxtacentromeric SAT transcripts has been reported in cancer, and whether SAT deregulation actively drives genomic instability in cancer or is merely a consequence of it remains to be shown [26]. In our study, the use of human tissue also limits us from concluding whether SAT transcripts contribute to progression of heart failure. It is also unclear at this time whether only a specific cell type of the heart is responsible for repeat element expression. Similarly, other confounding factors that are characteristic of studies like ours also exist, including medications that patients were on, the presence of other disease co-morbidities or other disease risk factors. Despite all these limitations, it is very striking to find that SAT transcripts alone, and not other repeat elements, are very highly upregulated in diseased hearts. Whatever their origin, their possible role in disease progression now warrants urgent investigation. As in cancer, SAT expression may highlight a potential link between genomic damage and heart failure disease progression. In end-stage diseased hearts, we have certainly observed significant and widespread DNA damage [29] that is out of proportion to the diminishingly low levels of myocyte cell death usually detected in end-stage failing hearts [30]. Furthermore, juxtacentromeric hypomethylation and SAT transcript abundance may indeed be related to the observation of polyploidy in diseased human myocytes [31].

## Conclusions

Our genome-wide analysis of repetitive element methylation in the cardiac genome has revealed a differential methylation profile in SAT repetitive elements, and possibly SINE1/7SL, but not other repeat families. SAT element hypomethylation was associated with significant upregulation of juxtacentromeric SAT transcripts in diseased hearts compared to healthy controls. The functional effect of these findings in cardiomyopathy remains to be demonstrated but the fundamental role of SAT non-coding transcripts in other contexts implies that this now merits further investigation.

## Materials and methods

### Human myocardial samples

Human LV myocardial tissue was collected under a protocol approved by the Papworth Hospital Tissue Bank Review Board and the Cambridgeshire South Research Ethics Committee, UK. Written and informed consent was obtained from patients undergoing cardiac transplantation for end-stage heart failure, including both ischemic and idiopathic cardiomyopathy (male Caucasians, aged 42 to 68 years). In our previous assessment of genome-wide DNA methylation using similar end-stage cardiomyopathic human hearts, methylation profiles and gene expression did not differ between ischemic and idiopathic cardiomyopathic hearts [10,12]. Others have similarly described the convergent pattern of gene expression in end-stage ischemic and dilated cardiomypathic human hearts [11,32]. We therefore used all ischemic and idiopathic cardiomyopathic samples collectively as representative of end-stage cardiomyopathy (EsCM). Normal LV tissues (CTRL) were from healthy male individuals (UK Human Tissue Bank, de Montfort University, UK). These were individuals with no prior clinical history of cardiovascular disease, diabetes mellitus or other forms of metabolic disease, and were not known to be on any long-term medications. CTRL LV tissue came from individuals who died from road traffic accidents except for one sample that came from an individual who suffered from hypoxic brain injury secondary to drowning. All CTRL LV samples were inspected at the time of post-mortem and any significant degree of coronary artery disease or myocardial disease was excluded. At the time of transplantation or cardiac harvest, whole hearts were removed after preservation and transported as previously described [29,33]. After analysis by a cardiovascular pathologist (MG), LV segments were cut and immediately stored in RNAlater (Ambion, Applied Biosystems, Warrington, UK). Individual LV sample details are listed in Additional file 13.

### Genomic DNA isolation

Genomic DNA (gDNA) was isolated from LV tissue as previously described [12]: 200 mg tissue was homogenized in G2 lysis buffer containing 80 mg/ml of RNase A with a handheld homogenizer (Polytron, VWR, Leics, UK), and proteinase K was added to a final concentration of 1 mg/ml and incubated at 50°C for at least 2 hours while rotating until all the tissue was fully digested. gDNA was purified with ×2 phenol:chloroform isolation and chloroform wash and precipitated with sodium chloride. After another wash with 70% ethanol, samples were quantified on a Qubit (Invitrogen, Paisley, UK).

### RNA isolation and cDNA synthesis

RNA was extracted from LV tissue by homogenizing at least 30 mg of frozen tissue in 0.5 ml of TRIreagent (Sigma-Aldrich, St Louis, MO, USA) with a handheld homogenizer (Polytron). Homogenates were centrifuged at 3,000 rpm for 3 minutes; supernatant was transferred to a clean Eppendorf; and RNA extraction was performed according to the manufacturer's protocol with the following modification. After chloroform extraction, ethanol was added to samples to a final concentration of 35%, and samples were loaded onto PureLink RNA columns (Invitrogen, 12183-018A). On-column DNase treatment was carried out with elution of the RNA. Integrity of all RNA samples was checked with the 2100 Bioanalyser (Agilent Technologies, Berks, UK). cDNA (20 μl) was synthesized from 1 mg total RNA using a mixture of both oligo-dT and random hexamers and the Superscript-III first-strand cDNA synthesis kit (Invitrogen).

### MeDIP-seq and H3K36me3 ChIP-seq datasets

Datasets for MeDIP followed by high-throughput sequencing (MeDIP-seq) and H3K36me3 ChIP-seq are as previously published [10].

### Quantitative PCR

To examine RNA transcript abundance of selected repetitive elements, real-time qPCR for myocardial cDNA was performed with 3 ml of 1:20 prediluted cDNA in a 12 μl reaction using SYBER greenER universal (Invitrogen, 11762100). To detect possible expansion of repetitive elements in the cardiac genome, real-time qPCR for myocardial gDNA samples was performed with 50 pg of gDNA in 12 μl reaction using SYBER greenER universal (Invitrogen, 11762100). The three candidate repeat sequences (*ALR*, *ALR_*, *ALRb*) were used for qPCR to validate possible difference between normal and diseased hearts. The primer sequences for each of the three sequences are shown in Additional file 16. qPCR for cDNA was normalized by a normalization factor generated for each sample with geNorm [34] based on expression relative to the housekeeping genes *RPLPO *and *TBP*. qPCR for gDNA was normalized to the abundance of control genomic loci (*OXT *and *GAPDH *promoters) where duplication or copy-number variation of these genomic loci is unlikely. PCR reactions were performed at least in triplicate and on the same diluted gDNA and cDNA samples. Reaction conditions were: 10 minutes at 95°C, 1 cycle; 15 s at 95°C, 60 s at 60°C, 41 cycles. Melt curves of the amplified products were used to verify that a single amplicon was generated with each PCR reaction.

### DNA repeat data preprocessing

The DNA repeat sequences were download from Repbase update version 16.7 [13]. The 1,166 repeats annotated as human were extracted and a pseudo-human repetitive sequence database was created.

### Short read alignments

The 36 bp single-end short read sequences of four normal and four diseased hearts were aligned against human reference genome assembly version hg18 (with repetitive regions masked out) [35,36] using Bowtie short read alignment software version 0.12.7 [37]. These short read samples were also aligned against the pseudo-human repeats database. Of both the datasets, only unique alignments were kept. The alignments with more than two mismatches were discarded. Repeat sequences having mean read coverage of <10 reads across both normal samples and diseased samples were eliminated due to lack of coverage, leaving 412 repeat sequences for further analyses.

### Differentially methylated repeats and repeat families

The number of reads aligned against the repeat sequences and human reference genome assembly were scaled to 15,000,000 to normalize the effect of the unequal number of reads generated by the MeDIP-seq experiment. The total number of reads generated by the experiment is shown in Additional file 2. To compare a pair of normal and diseased samples, we computed odds ratio using Fisher's exact test. The approach is described in the context of MeDIP-seq data analysis by Bock *et al. *[38]. The normal versus diseased pair of samples were compared for each repeat sequence. Furthermore, all repeats belonging to the same family were merged, resulting in the total number of aligned reads in a repeat family. The number of reads per family between normal and diseased samples was subsequently compared using the Fisher's exact test (Additional file 4). The same grouping was applied to repeat classes followed by classwise Fisher's exact test. The repeats, repeat families and repeat classes in which more than two pairs (CTRL versus EsCM) had an insignificant Fisher's *P*-value (*P *> 0.05) were removed from further analyses. The two groups were also compared using unpaired Welch's *t*-test for each of the 412 repeat elements. The *P*-values were adjusted for multiple comparisons using the Benjamini and Hochberg method (Additional file 7).

## Abbreviations

ChIP: chromatin immunoprecipitation; CTRL: control; DMRep: differentially methylated repetitive element; EsCM: end-stage cardiomyopathic; gDNA: genomic DNA; H3K36me3: tri-methylated histone H3 at lysine 36; LINE: long interspersed nuclear element; LV: left ventricular; meDIP: methylated DNA immunoprecipitation; qPCR: quantitative PCR; SAT: satellite; SINE: short interspersed nuclear element.

## Competing interests

The authors declare that they have no competing interests.

## Authors' contributions

SH carried out the computational analyses and co-wrote the manuscript. LC, ER, LS and AV carried out molecular genetic studies. MM performed the original MeDIP experiments. MKC performed the density plot analysis. MG analyzed and archived the human tissue. RF conceived of the study and co-wrote the manuscript. All authors read and approved the final manuscript for publication.

## Supplementary Material

Additional file 1 **Figure S1 - schematic view of the analysis workflow**. **(a) **Methylated DNA immunoprecipitation (MeDIP) was conducted to isolate methylated DNA fragments across four end-stage cardiomyopathic (EsCM 1 to 4) and four normal healthy control (CTRL A to D) hearts as listed in Additional file 2 and as published [10]. **(b) **MeDIP samples were sequenced using an Illumina genome analyzer (GIIx). **(a) **Short single-end reads from high-throughput sequencing were aligned against the human reference genome assembly (Hg18) and repeats database (Repbase). **(d) **Number of unique reads was normalized with reference to the respective total number of reads generated for each sample, and used as a proxy for the level of methylation for all repeat sequences. **(e) **Differential methylation between each of EsCM and CTRL samples was compared using Fisher's exact test statistic as well as unpaired Welch's *t*-test. **(f) **Differentially methylated repeat elements (DMReps) were selected for downstream analysis. Click here for file

Additional file 2 **Table S1 - number of sequencing reads from LV samples**. Click here for file

Additional file 3 **Figure S2 - fully annotated large-scale version of Figure **1. Click here for file

Additional file 4 **List of all annotated repeat elements in the human genome**. Click here for file

Additional file 5 **Figure S3**. **(a) **All EsCM LV samples (EsCM 1 to 4) were compared against each of the CTRL samples (CTRL 1 to 4) using Fisher's exact test (*P *< 0.05 in at least 14 comparisons). Green color indicates hypomethylation in EsCM compared to the corresponding CTRL and red color indicates the converse, hypermethylation in EsCM. The color bar on the vertical axis represents families of repeat elements. A consistent pattern of hypomethylation was found only in satellite (SAT) family repeats in EsCM (arrow labels). **(b) **A bar chart representing the number of repeat sequences per family, following the elimination of repeats that were not differentially methylated between the two groups of samples. Click here for file

Additional file 6 **Figure S4 - fully annotated version of Additional file **5. Click here for file

Additional file 7 **CTRL versus EsCM comparison of each repeat element's methylation using unpaired Welch's *t*-test**. Click here for file

Additional file 8 **Figure S5 - count data of repeat sequences merged into respective families**. All EsCM LV samples (EsCM 1 to 4) were compared against each of the CTRL samples (CTRL 1 to 4) using Fisher's exact test (*P *< 0.05 in at least 14 comparisons). Green color indicates hypomethylation in EsCM compared to the corresponding CTRL and red color indicates the converse, hypermethylation in EsCM. The color bar on the vertical axis represents families of repeat elements. A consistent pattern of hypomethylation was found only in satellite (SAT) family repeats in EsCM. Click here for file

Additional file 9 **Figure S6 - the count data of repeat sequences merged into respective classes**. All EsCM LV samples (EsCM 1 to 4) were compared against each of the CTRL samples (CTRL 1 to 4) using Fisher's exact test (*P *< 0.05 in at least 14 comparisons). Green color indicates hypomethylation in EsCM compared to the corresponding CTRL and red color indicates the converse, hypermethylation in EsCM. The color bar on the vertical axis represents families of repeat elements. A consistent pattern of hypomethylation was found only in satellite (SAT) family repeats in EsCM. Click here for file

Additional file 10 **List of all *ALR*, *ALR_ and ALRb *elements and coordinates in the human genome according to Hg18**. Click here for file

Additional file 11 **Figure S7**. **(a-c) **Average density plot for the methylation of *ALR *(a), *ALR*_ (b) and *ALRb *(c) comparing between EsCM (red) and CTRL (blue). Methylation density was consistently reduced in EsCM within the global coordinates of each repeat element (represented collectively here as 0.0 to 1.0 on the X-axis) as well as extending to the flanks (+3.0 and -3.0 kb) of the repeat elements. Light blue- and cream-colored error bars represent Bayesian credible intervals for CTRL and EsCM, respectively. See Movassagh *et al. *and Down *et al. *for detailed methods of methylation density analysis [10,39]. Click here for file

Additional file 12 **Figure S8 - quantitative PCR using genomic DNA for the copy number abundance of SAT family repeat sequences (*ALR*, *ALR*_ and *ALRb*)**. **(a-c) **Quantification of copy number abundance for *ALR *(a), *ALR*_ (b) and *ALRb *(c) repeat elements was performed for EsCM and CTRL LV samples (EsCM A to H and CTRL 1 to 16), and normalized to the copy number for a control genomic locus (promoter region of *OXT*). A similar result was obtained when normalized to a second control genome locus (promoter region of *GAPDH*). The significance of difference between the two groups was computed using unpaired Wilcoxon rank-sum test, and a significance of *P *< 0.05 was detected only for *ALRb*. Click here for file

Additional file 13 **Table S2 - list of LV sample details**. Click here for file

Additional file 14 **Figure S9**. **(a-c) **Average density plot for the H3K36me3 ChIP-seq enrichment of *ALR *(a), *ALR*_ (b) and *ALRb *(c) comparing between EsCM (red) and CTRL (blue), similar to Additional file 11. H3K36me3 demarcates genomic regions that are actively transcribed. An enrichment of H3K36me3 mark in all three repeat elements in EsCM is consistent with increased transcriptional activity at these sites in EsCM. Click here for file

Additional file 15 **Table S3 - genes related to SAT elements**. Click here for file

Additional file 16 **Table S4 - primers used for quantification PCR for SAT repeat elements**. Click here for file
