# Supplementary figures and images for: The landscape of DNA repeat elements in human heart failure
Source: Genome Biol. 2012 Oct 3;13(10):R90. doi: 10.1186/gb-2012-13-10-r90 (PMC3491418; doi:10.1186/gb-2012-13-10-r90)

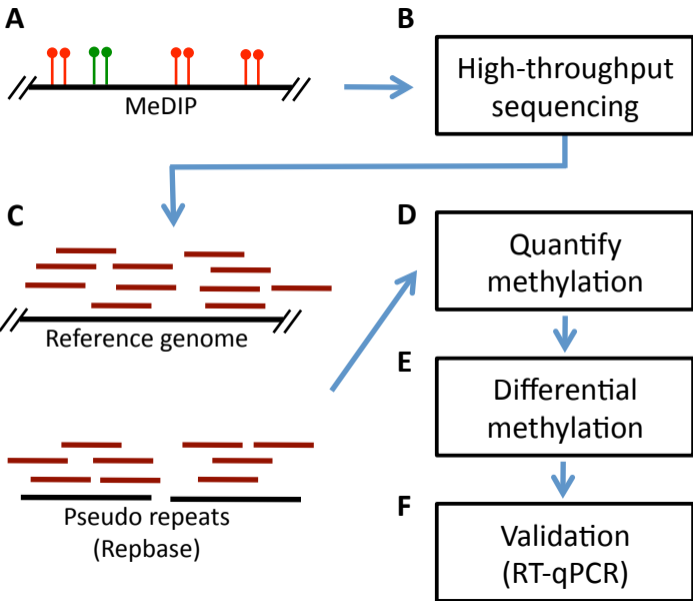

Supplement: Additional file 1 — Figure S1 - schematic view of the analysis workflow. (a) Methylated DNA immunoprecipitation (MeDIP) was conducted to isolate methylated DNA fragments across four end-stage cardiomyopathic (EsCM 1 to 4) and four normal healthy control (CTRL A to D) hearts as listed in Additional file 2 and as published [10]. (b) MeDIP samples were sequenced using an Illumina genome analyzer (GIIx). (a) Short single-end reads from high-throughput sequencing were aligned against the human reference genome assembly (Hg18) and repeats database (Repbase). (d) Number of unique reads was normalized with reference to the respective total number of reads generated for each sample, and used as a proxy for the level of methylation for all repeat sequences. (e) Differential methylation between each of EsCM and CTRL samples was compared using Fisher's exact test statistic as well as unpaired Welch's t-test. (f) Differentially methylated repeat elements (DMReps) were selected for downstream analysis. [file gb-2012-13-10-r90-S1.pdf]

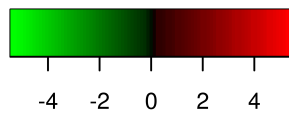

**A**

DMRep ( $\log_2\text{OR}$ )

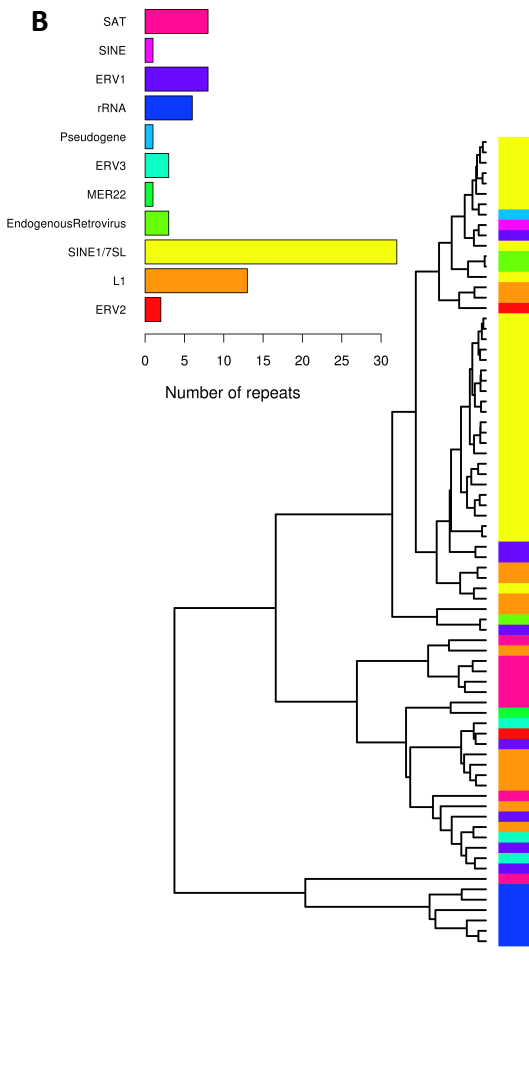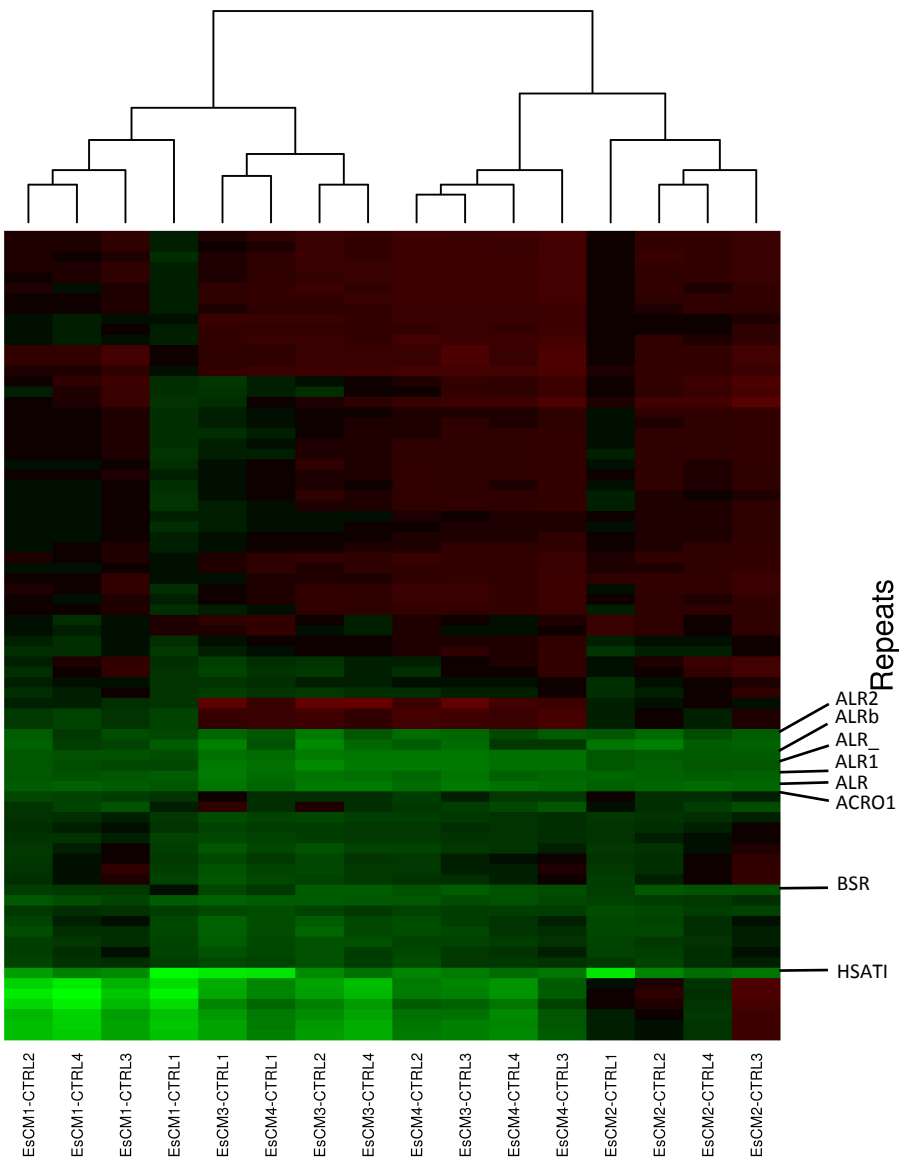

Supplement: Additional file 5 — Figure S3. (a) All EsCM LV samples (EsCM 1 to 4) were compared against each of the CTRL samples (CTRL 1 to 4) using Fisher's exact test (P < 0.05 in at least 14 comparisons). Green color indicates hypomethylation in EsCM compared to the corresponding CTRL and red color indicates the converse, hypermethylation in EsCM. The color bar on the vertical axis represents families of repeat elements. A consistent pattern of hypomethylation was found only in satellite (SAT) family repeats in EsCM (arrow labels). (b) A bar chart representing the number of repeat sequences per family, following the elimination of repeats that were not differentially methylated between the two groups of samples. [file gb-2012-13-10-r90-S5.pdf]

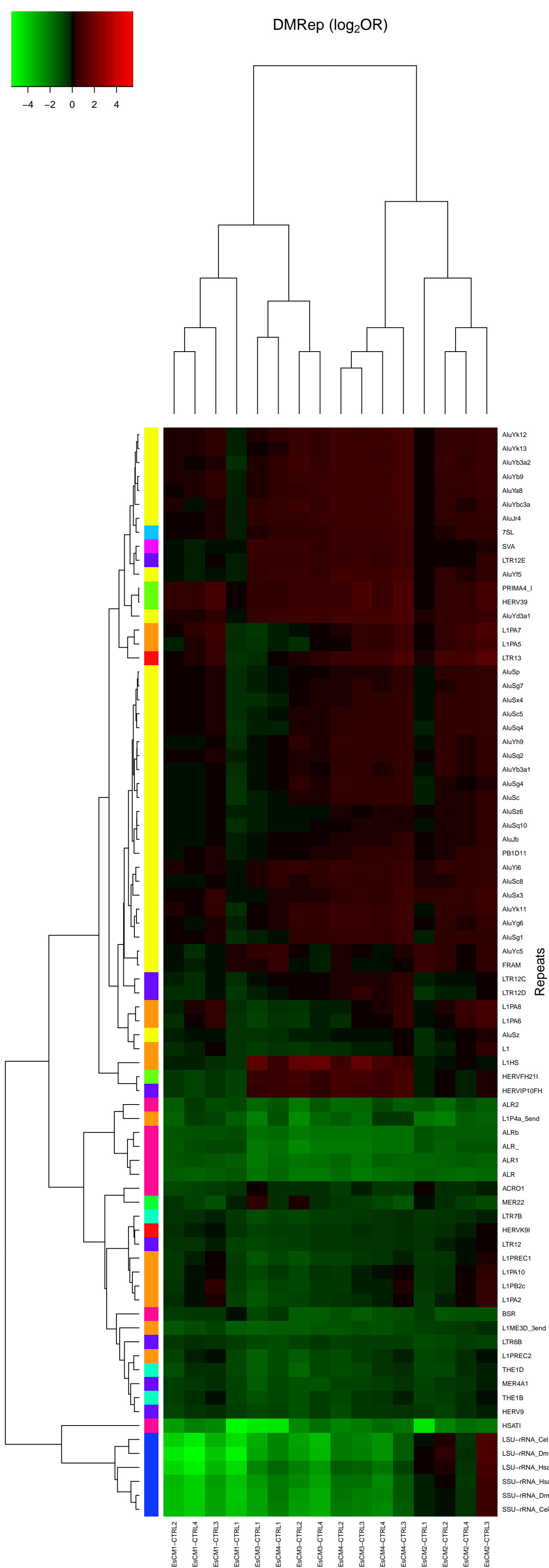

Supplement: Additional file 6 — Figure S4 - fully annotated version of Additional file 5. [file gb-2012-13-10-r90-S6.pdf]

A

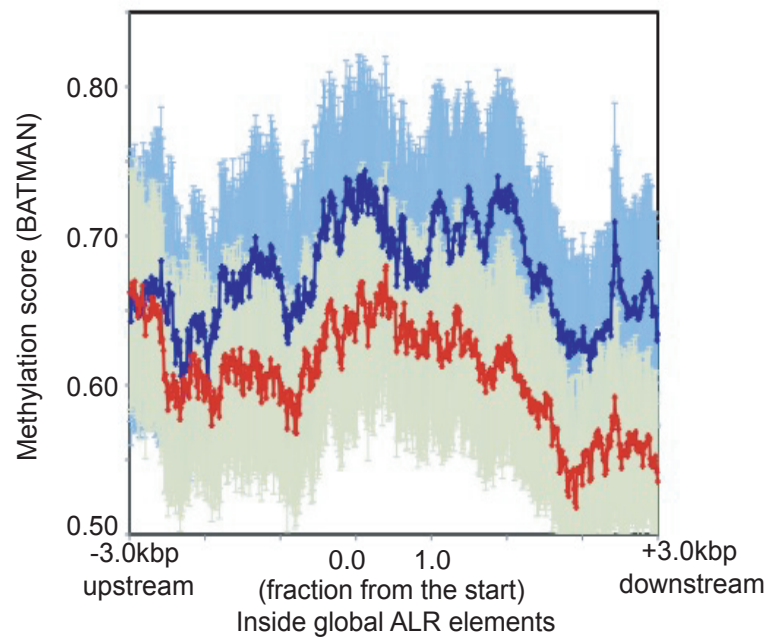

B

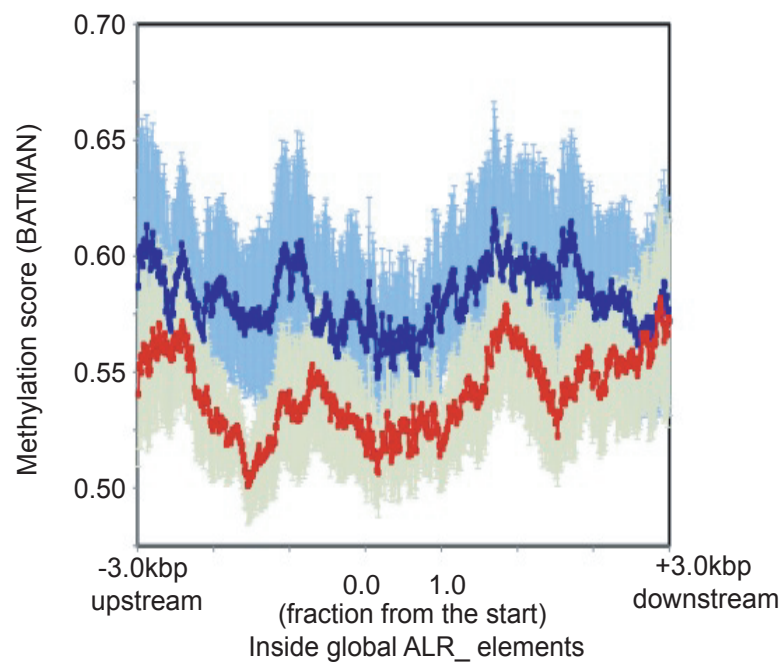

C

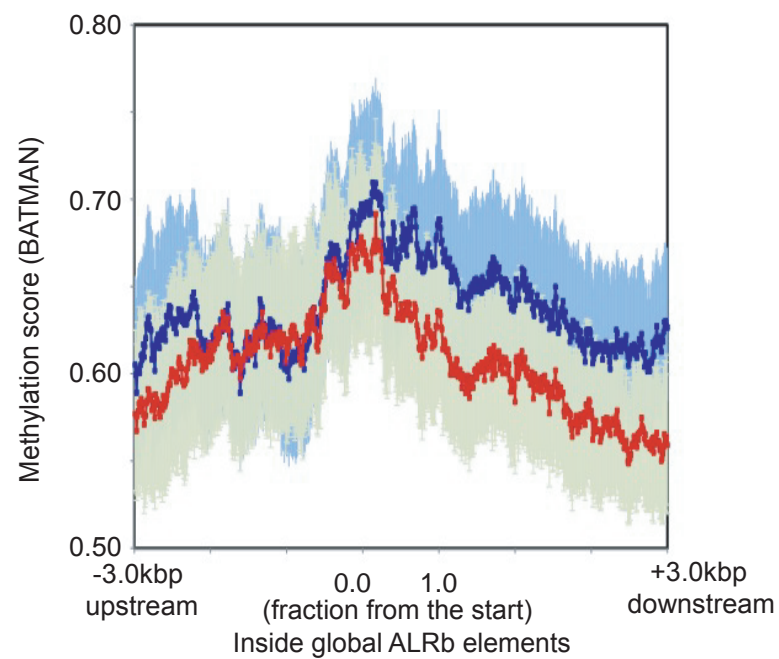

Supplement: Additional file 11 — Figure S7. (a-c) Average density plot for the methylation of ALR (a), ALR_ (b) and ALRb (c) comparing between EsCM (red) and CTRL (blue). Methylation density was consistently reduced in EsCM within the global coordinates of each repeat element (represented collectively here as 0.0 to 1.0 on the X-axis) as well as extending to the flanks (+3.0 and -3.0 kb) of the repeat elements. Light blue- and cream-colored error bars represent Bayesian credible intervals for CTRL and EsCM, respectively. See Movassagh et al. and Down et al. for detailed methods of methylation density analysis [10,39]. [file gb-2012-13-10-r90-S11.pdf]

A

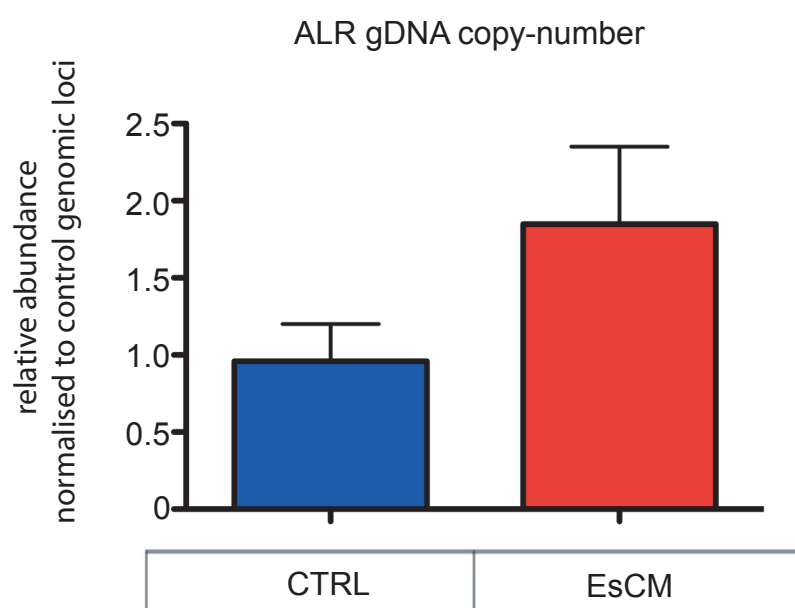

B

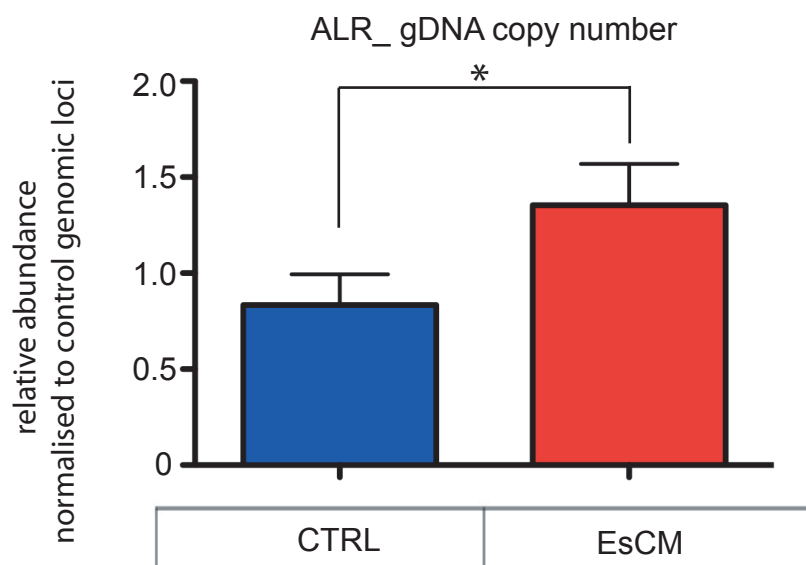

C

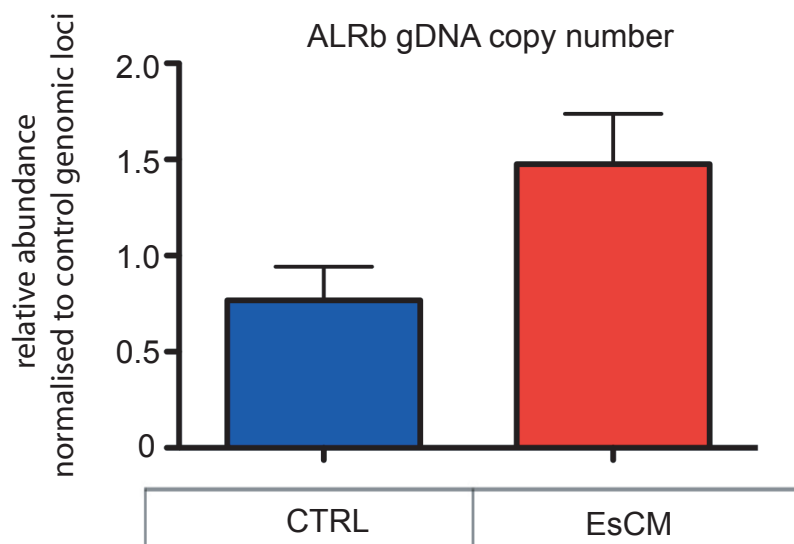

Supplement: Additional file 12 — Figure S8 - quantitative PCR using genomic DNA for the copy number abundance of SAT family repeat sequences (ALR, ALR_ and ALRb). (a-c) Quantification of copy number abundance for ALR (a), ALR_ (b) and ALRb (c) repeat elements was performed for EsCM and CTRL LV samples (EsCM A to H and CTRL 1 to 16), and normalized to the copy number for a control genomic locus (promoter region of OXT). A similar result was obtained when normalized to a second control genome locus (promoter region of GAPDH). The significance of difference between the two groups was computed using unpaired Wilcoxon rank-sum test, and a significance of P < 0.05 was detected only for ALRb. [file gb-2012-13-10-r90-S12.pdf]

A

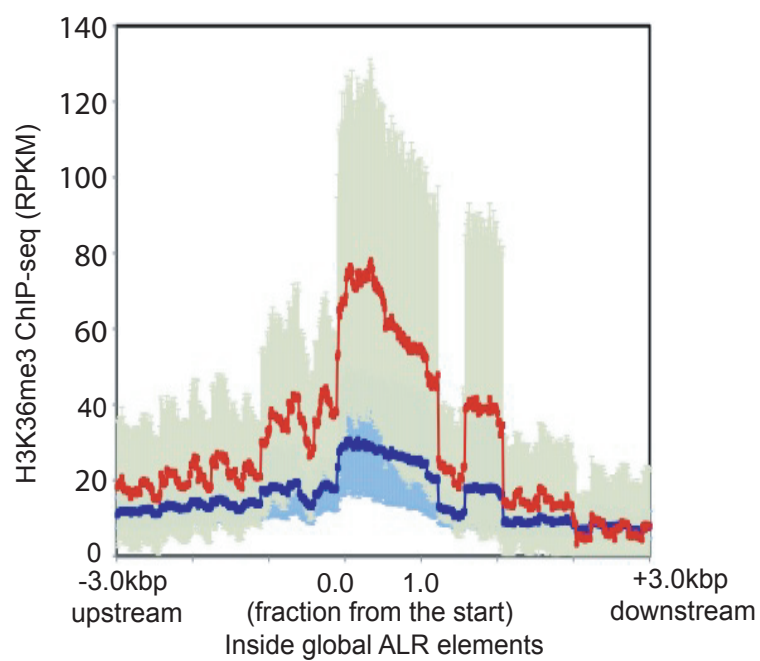

B

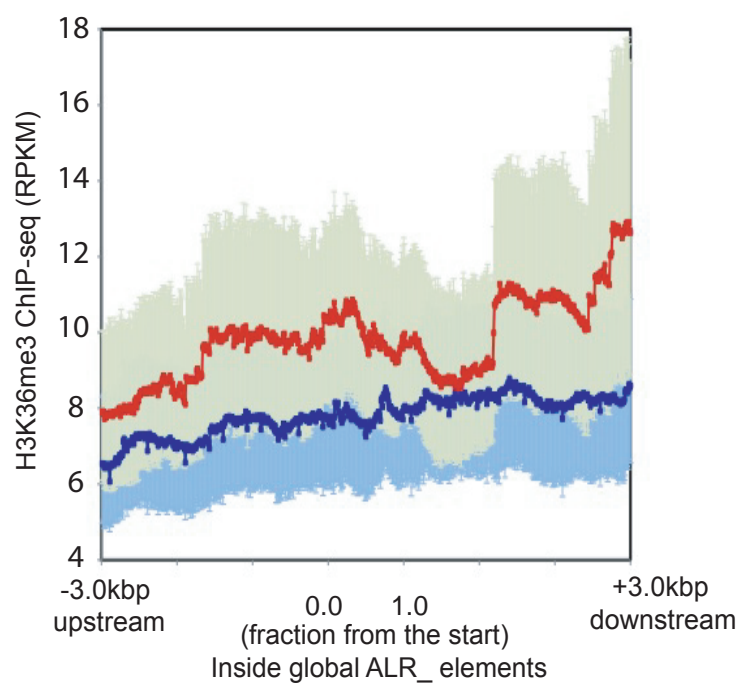

C

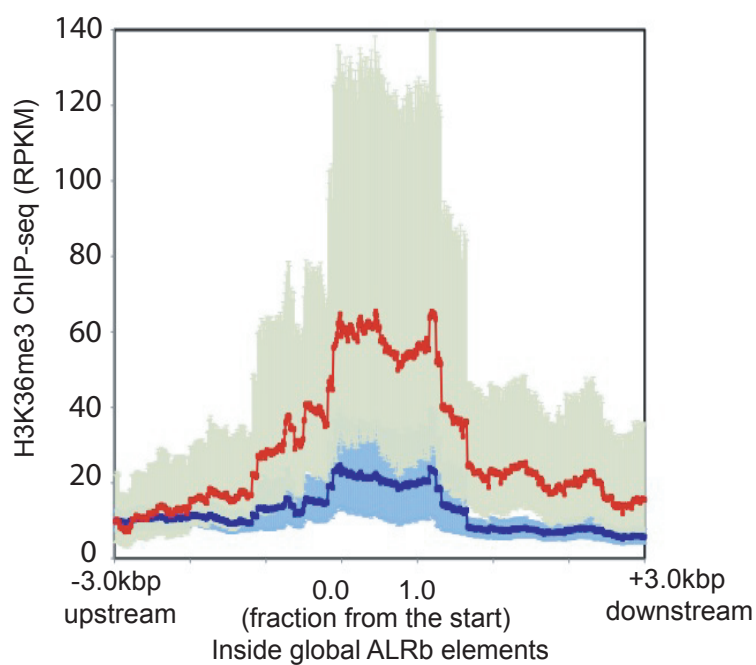

Supplement: Additional file 14 — Figure S9. (a-c) Average density plot for the H3K36me3 ChIP-seq enrichment of ALR (a), ALR_ (b) and ALRb (c) comparing between EsCM (red) and CTRL (blue), similar to Additional file 11. H3K36me3 demarcates genomic regions that are actively transcribed. An enrichment of H3K36me3 mark in all three repeat elements in EsCM is consistent with increased transcriptional activity at these sites in EsCM. [file gb-2012-13-10-r90-S14.pdf]
