# Supplementary material for: The landscape of DNA repeat elements in human heart failure
Source: Genome Biol. 2012 Oct 3;13(10):R90. doi: 10.1186/gb-2012-13-10-r90 (PMC3491418; doi:10.1186/gb-2012-13-10-r90)
Supplement: Additional file 2 — Table S1 - number of sequencing reads from LV samples. [file gb-2012-13-10-r90-S2.docx]

# Supplementary Table 1. Number of sequencing reads from LV samples

| **LV Sample** | **Total number of reads** | **Normalised number of uniquely mapped reads against Hg18** |
| --- | --- | --- |
| CTRL A | 14,284,037 | 6,170,830 |
| CTRL B | 15,866,405 | 6,378,017 |
| CTRL C | 16,729,277 | 6,451,329 |
| CTRL D | 15,099,359 | 6,248,898 |
| EsCM 1 | 15,792,474 | 6,124,398 |
| EsCM 2 | 15,545,003 | 5,500,468 |
| EsCM 3 | 18,564,406 | 6,987,639 |
| EsCM 4 | 15,161,014 | 6,865,064 |
