# Supplementary material for: The landscape of DNA repeat elements in human heart failure
Source: Genome Biol. 2012 Oct 3;13(10):R90. doi: 10.1186/gb-2012-13-10-r90 (PMC3491418; doi:10.1186/gb-2012-13-10-r90)
Supplement: Additional file 4 — List of all annotated repeat elements in the human genome. [file gb-2012-13-10-r90-S4.pdf]

			Normal				Diseased (Dilated)		Diseased (Ischaemic)	
**Repeat**	**Family**	**Class**	**CTRL1** (s01)	**CTRL2** (s02)	**CTRL3** (S03)	**CTRL4** (s04)	**EsCM1** (s06)	**EsCM2** (s07)	**EsCM3** (s12)	**EsCM4** (s13)
HERVK	ERV2	LTR	1725	1480	1513	1497	1456	1329	1517	1630
HERVK11DI	ERV2	LTR	84	78	54	65	65	55	69	49
HERVK11I	ERV2	LTR	352	392	343	327	317	282	288	326
HERVK13I	ERV2	LTR	35	24	17	16	20	33	21	25
HERVK22I	ERV2	LTR	272	276	202	238	212	186	181	197
HERVK3I	ERV2	LTR	50	69	54	61	47	52	46	56
HERVK9I	ERV2	LTR	913	865	697	764	644	595	591	608
HERVKC4	ERV2	LTR	36	25	30	36	21	21	19	19
LTR13	ERV2	LTR	1275	900	648	830	902	1323	1130	1440
LTR13A	ERV2	LTR	667	515	328	437	400	511	460	589
LTR14	ERV2	LTR	27	24	35	34	28	24	19	19
LTR14A	ERV2	LTR	151	142	114	131	140	146	133	188
LTR14B	ERV2	LTR	125	122	106	105	99	129	86	156
LTR14C	ERV2	LTR	129	107	78	87	92	80	77	94
LTR22	ERV2	LTR	30	28	20	31	24	29	32	28
LTR22A	ERV2	LTR	36	40	32	31	27	43	40	36
LTR22B1	ERV2	LTR	14	13	13	15	11	14	15	16
LTR22C0	ERV2	LTR	26	23	11	22	11	18	13	23
LTR3	ERV2	LTR	15	22	15	12	16	23	23	26
LTR3A	ERV2	LTR	86	86	64	77	74	96	74	89
LTR3B	ERV2	LTR	93	69	75	75	73	75	61	63
LTR5	ERV2	LTR	1686	1673	1459	1541	1403	1317	1330	1518
LTR5A	ERV2	LTR	366	340	285	351	261	250	281	284
LTR5B	ERV2	LTR	985	880	770	930	885	887	972	991
LTR5_Hs	ERV2	LTR	1993	1998	1797	1918	1912	1930	1724	1895
MER11A	ERV2	LTR	665	644	561	539	474	567	481	519
MER11B	ERV2	LTR	879	730	620	696	607	663	647	724
MER11C	ERV2	LTR	816	751	589	688	563	596	526	655
MER11D	ERV2	LTR	427	405	282	302	232	285	259	302
MER9	ERV2	LTR	76	49	55	55	42	43	43	35
MER9B	ERV2	LTR	46	38	35	36	34	48	36	46
MER9a1	ERV2	LTR	393	341	295	313	273	267	239	294
MER9a2	ERV2	LTR	142	127	105	120	85	97	74	80
MER9a3	ERV2	LTR	202	235	181	203	180	206	162	162
L1	L1	LINE	68467	64639	49958	56691	50583	51048	49236	55058
L1HS	L1	LINE	97388	89059	92903	82389	72874	73310	284243	158769
L1M1B_5	L1	LINE	15	17	6	2	10	9	8	12
L1M1_5	L1	LINE	291	361	169	219	189	169	163	204
L1M2C_5	L1	LINE	25	31	13	21	15	19	14	21
L1M2_5	L1	LINE	67	57	52	50	42	41	40	43
L1M4B	L1	LINE	17	14	10	12	10	14	6	12
L1MA1	L1	LINE	338	287	138	219	165	171	132	195
L1MA10	L1	LINE	34	26	10	12	18	15	9	13
L1MA2	L1	LINE	142	130	74	110	91	95	95	92
L1MA3	L1	LINE	125	143	86	92	65	85	53	59
L1MA4	L1	LINE	327	311	187	169	167	147	118	160
L1MA4A	L1	LINE	294	315	186	209	165	169	129	206

L1MA5	L1	LINE	151	150	94	95	79	71	59	114
L1MA5A	L1	LINE	243	245	124	150	131	130	87	155
L1MA6	L1	LINE	258	212	129	132	153	118	86	169
L1MA7	L1	LINE	120	123	68	84	91	70	60	76
L1MA8	L1	LINE	133	146	74	80	85	63	59	81
L1MA9	L1	LINE	70	83	46	57	61	49	42	57
L1MB1	L1	LINE	58	72	46	50	42	46	44	44
L1MB2	L1	LINE	182	167	89	87	110	80	74	99
L1MB3	L1	LINE	308	292	221	222	207	179	158	230
L1MB3_5	L1	LINE	16	8	9	14	11	17	6	12
L1MB4	L1	LINE	103	92	78	64	59	60	48	54
L1MB5	L1	LINE	76	77	55	65	56	58	41	43
L1MB7	L1	LINE	44	46	36	26	20	24	11	19
L1MB8	L1	LINE	59	62	53	46	43	34	31	37
L1MC1	L1	LINE	153	173	111	115	102	94	82	111
L1MC2	L1	LINE	71	72	38	41	47	51	37	36
L1MC3	L1	LINE	37	39	20	17	18	22	21	21
L1MCA_5	L1	LINE	37	35	21	23	17	26	16	26
L1MD1	L1	LINE	20	19	14	12	14	12	11	17
L1MD2	L1	LINE	45	37	16	20	20	15	13	22
L1MDA_5	L1	LINE	16	14	7	11	6	13	6	8
L1ME1	L1	LINE	40	38	17	27	22	23	20	19
L1ME3D_3end	L1	LINE	60	57	44	49	23	30	23	24
L1ME_ORF2	L1	LINE	368	358	225	235	188	204	121	176
L1P4a_5end	L1	LINE	81	94	56	52	31	17	18	39
L1P4e_5end	L1	LINE	15	13	14	9	12	9	16	8
L1PA10	L1	LINE	3443	3485	2195	2459	2245	2351	2015	2513
L1PA11	L1	LINE	1216	1164	725	841	767	856	766	885
L1PA12	L1	LINE	512	410	294	353	388	438	343	421
L1PA12_5	L1	LINE	116	110	82	75	69	50	69	86
L1PA13	L1	LINE	805	727	455	534	556	668	494	659
L1PA13_5	L1	LINE	131	110	76	91	75	64	66	75
L1PA14	L1	LINE	940	874	578	625	560	581	462	583
L1PA14_5	L1	LINE	21	17	16	13	9	9	12	14
L1PA15	L1	LINE	982	873	563	619	540	544	422	576
L1PA16	L1	LINE	891	890	528	594	535	509	359	535
L1PA16_5	L1	LINE	225	245	139	149	122	104	97	134
L1PA2	L1	LINE	4863	3546	2465	2927	2644	2745	2216	2815
L1PA3	L1	LINE	3538	4216	2416	3179	3353	4102	2720	3760
L1PA4	L1	LINE	3718	3690	2320	3003	3199	3881	3278	4144
L1PA5	L1	LINE	5483	5040	3182	4024	4341	5164	4500	5558
L1PA6	L1	LINE	12525	11385	6984	8595	8984	10393	8150	10180
L1PA7	L1	LINE	5684	4508	3036	3870	4667	5403	4550	5328
L1PA7_5	L1	LINE	3494	3645	2737	2994	2701	2619	2497	2790
L1PA8	L1	LINE	5191	4737	3196	3723	4159	4623	3735	4515
L1PB1	L1	LINE	1040	899	534	714	671	711	560	716
L1PB2	L1	LINE	881	787	461	585	523	559	419	529
L1PB2c	L1	LINE	3659	3243	1986	2494	2337	2236	1862	2366
L1PB3	L1	LINE	287	277	171	193	162	152	110	170
L1PB4	L1	LINE	652	580	342	386	328	280	211	371

L1PBA1_5	L1	LINE	110	113	82	92	92	92	89	78
L1PBA_5	L1	LINE	331	374	224	257	206	193	183	223
L1PREC1	L1	LINE	26205	24952	16025	17953	15758	15405	11667	14767
L1PREC2	L1	LINE	8633	8353	5012	5631	4685	4146	3316	4509
L1P_MA2	L1	LINE	619	609	336	429	378	349	283	361
MER25	L1	LINE	69	69	31	52	36	37	40	37
TRNA_GLY	tRNA	tRNA	5	9	15	8	14	30	9	41
tRNA-Asp-GAY	tRNA	tRNA	1	8	7	4	8	20	6	50
tRNA-Leu-TTA(m)	tRNA	tRNA	59	41	47	28	26	30	19	29
AluJb	SINE1/7SL	SINE	37370	35015	31530	33487	31882	33592	39333	42049
AluJr	SINE1/7SL	SINE	349	306	288	290	348	319	364	433
AluJr4	SINE1/7SL	SINE	12428	11135	10026	10613	11171	11620	16897	18430
AluSc	SINE1/7SL	SINE	49882	40168	37244	40535	36641	39165	50035	53337
AluSc5	SINE1/7SL	SINE	68064	55257	51842	54006	56810	60607	69123	73023
AluSc8	SINE1/7SL	SINE	8166	8198	7540	7818	7545	8379	10551	11691
AluSg1	SINE1/7SL	SINE	7853	5886	5738	5720	6054	6072	8086	8726
AluSg4	SINE1/7SL	SINE	19389	15531	14940	15786	14313	14857	20454	21912
AluSg7	SINE1/7SL	SINE	31647	25912	23664	24941	25571	26238	30484	32388
AluSp	SINE1/7SL	SINE	102095	85261	79720	83689	84566	88586	101491	106704
AluSq	SINE1/7SL	SINE	10500	8997	8222	8194	7540	7697	8370	8792
AluSq10	SINE1/7SL	SINE	51048	46047	41461	43014	41827	43667	49329	52379
AluSq2	SINE1/7SL	SINE	41907	36209	35422	37566	36881	38416	47081	49471
AluSq4	SINE1/7SL	SINE	20481	15088	14763	14883	15512	16499	19541	20712
AluSx3	SINE1/7SL	SINE	2558	2362	1949	2291	2443	2746	2881	3210
AluSx4	SINE1/7SL	SINE	70994	58508	52040	54177	56938	60113	67036	71720
AluSz	SINE1/7SL	SINE	20450	17834	16159	16372	14741	15207	16634	17632
AluSz6	SINE1/7SL	SINE	92979	85791	81610	84793	80695	86949	93067	101578
AluYa8	SINE1/7SL	SINE	16251	13842	12032	13096	14147	15388	20955	23346
AluYb3a1	SINE1/7SL	SINE	82275	70725	69428	74928	67537	72539	91295	97607
AluYb3a2	SINE1/7SL	SINE	37005	27260	27405	30344	30705	33482	45931	48928
AluYb9	SINE1/7SL	SINE	40710	33328	30518	32340	36003	38716	52921	58377
AluYbc3a	SINE1/7SL	SINE	34153	27565	27566	30720	29485	31557	46861	49421
AluYc2	SINE1/7SL	SINE	4705	4082	3905	4060	4044	4337	5284	5605
AluYc5	SINE1/7SL	SINE	14307	17402	17846	19855	16189	18165	19518	21727
AluYd3a1	SINE1/7SL	SINE	3822	3169	2899	3131	3549	3755	5731	6180
AluYd8	SINE1/7SL	SINE	1731	2292	2322	2546	2012	2321	2485	2746
AluYe5	SINE1/7SL	SINE	2275	1680	1631	1800	1533	1634	3207	3660

AluYf2	SINE1/7SL	SINE	3154	2748	2681	2848	2676	2923	3954	4157
AluYf5	SINE1/7SL	SINE	2401	2237	2185	2357	2023	2306	3698	4065
AluYg6	SINE1/7SL	SINE	154723	127961	124036	133065	129375	138563	190408	203946
AluYh9	SINE1/7SL	SINE	144314	123740	116121	124575	118276	128039	161385	173691
AluYi6	SINE1/7SL	SINE	40214	35676	35120	37368	37347	40341	52024	55115
AluYk11	SINE1/7SL	SINE	182942	141822	133007	144951	150296	161402	208996	223940
AluYk12	SINE1/7SL	SINE	45652	36605	34766	36397	39900	43420	57166	62119
AluYk13	SINE1/7SL	SINE	136854	105316	99718	107422	115641	123254	167127	179753
FAM	SINE1/7SL	SINE	124	95	93	104	94	77	92	136
FLAM	SINE1/7SL	SINE	1232	946	899	875	799	777	833	917
FRAM	SINE1/7SL	SINE	16354	20012	20805	22202	18664	21145	21443	24119
PB1D11	SINE1/7SL	SINE	15875	15365	12907	13199	13987	14653	16987	18744
CHARLIE3	hAT	DNA	82	73	72	71	47	45	48	50
Charlie12	hAT	DNA	103	91	66	81	70	71	53	65
MER107	hAT	DNA	17	10	9	6	9	9	10	5
MER1A	hAT	DNA	76	64	49	38	34	43	28	47
MER1B	hAT	DNA	457	420	317	391	329	342	330	332
MER20	hAT	DNA	45	52	28	43	32	28	42	35
MER30	hAT	DNA	60	49	31	38	23	28	25	39
MER30B	hAT	DNA	27	27	17	31	16	16	15	19
MER75	DNAtransposon	DNA	46	39	35	35	33	24	23	22
MER82	DNAtransposon	DNA	16	10	6	11	8	6	7	11
MER85	DNAtransposon	DNA	20	23	22	17	9	12	13	15
GOLEM	Mariner/Tc1	DNA	17	9	8	12	9	4	9	10
GOLEM_B	Mariner/Tc1	DNA	29	24	19	20	23	23	16	33
GOLEM_C	Mariner/Tc1	DNA	19	14	12	13	18	18	8	16
HSMAR1	Mariner/Tc1	DNA	507	394	290	341	317	363	269	352
HSMAR2	Mariner/Tc1	DNA	80	74	45	54	47	34	27	41
MADE1	Mariner/Tc1	DNA	19	19	14	22	8	7	7	10
MER2	Mariner/Tc1	DNA	27	33	14	22	13	12	15	13
TIGGER1	Mariner/Tc1	DNA	653	611	407	455	400	370	268	398
TIGGER2	Mariner/Tc1	DNA	150	106	50	82	86	101	115	137
TIGGER5	Mariner/Tc1	DNA	26	22	22	18	25	13	9	20
ZOMBI	Mariner/Tc1	DNA	50	67	26	32	28	27	24	17
ZOMBI_B	Mariner/Tc1	DNA	16	9	14	14	7	6	6	7
HERV15I	EndogenousRetrovirus	LTR	90	63	62	68	68	61	57	66

HERV18	EndogenousRetrovirus	LTR	98	113	75	75	65	77	65	84
HERV1_LTR	EndogenousRetrovirus	LTR	18	12	8	14	9	8	8	15
HERV30I	EndogenousRetrovirus	LTR	148	166	109	121	85	83	92	88
HERV39	EndogenousRetrovirus	LTR	24340	20117	15014	19940	25540	23284	33181	32721
HERV46I	EndogenousRetrovirus	LTR	51	61	59	71	47	34	42	40
HERVFH19I	EndogenousRetrovirus	LTR	109	101	84	102	105	95	87	75
HERVFH21I	EndogenousRetrovirus	LTR	1424	1205	1204	1472	813	1127	2275	2398
HERVH48I	EndogenousRetrovirus	LTR	921	797	834	870	738	708	814	811
HERVP71A_I	EndogenousRetrovirus	LTR	85	83	63	65	55	44	43	67
HUERS-P2	EndogenousRetrovirus	LTR	26	24	16	20	9	17	17	17
HUERS-P3B	EndogenousRetrovirus	LTR	40	53	44	42	27	22	18	18
MER31	EndogenousRetrovirus	LTR	11	13	10	14	9	11	10	8
MER41I	EndogenousRetrovirus	LTR	15	18	14	16	9	6	6	10
MER4BI	EndogenousRetrovirus	LTR	32	26	25	15	14	14	12	13
MER57A_I	EndogenousRetrovirus	LTR	37	40	26	30	14	16	27	25
MER57I	EndogenousRetrovirus	LTR	89	78	51	60	47	44	43	72
MER61A	EndogenousRetrovirus	LTR	25	22	15	10	17	15	11	14
MER84I	EndogenousRetrovirus	LTR	15	20	12	14	13	13	19	16
PRIMA41	EndogenousRetrovirus	LTR	78	80	66	75	65	42	36	38
PRIMA4_I	EndogenousRetrovirus	LTR	57400	47487	36033	47054	61179	55143	79059	77543
MER22	MER22	LTR	14468	17655	30314	22479	12549	12734	21133	13013
ERVL	ERV3	LTR	184	191	97	122	117	109	76	112
ERVL-B4	ERV3	LTR	289	258	165	185	162	175	174	185
ERVL-E	ERV3	LTR	70	74	53	53	49	34	35	43
HERVL	ERV3	LTR	2186	1937	1210	1315	1213	1135	1023	1259
HERVL66I	ERV3	LTR	64	46	41	59	33	40	34	46
LTR18A	ERV3	LTR	41	44	42	51	41	40	31	36

LTR18B	ERV3	LTR	49	33	34	37	28	32	27	27
LTR18C	ERV3	LTR	19	29	22	17	22	19	23	21
LTR19A	ERV3	LTR	28	41	23	22	19	18	15	25
LTR32	ERV3	LTR	27	26	18	19	20	22	14	24
LTR66	ERV3	LTR	67	61	48	46	37	54	32	49
LTR7A	ERV3	LTR	1282	1221	911	1045	767	718	907	990
LTR7B	ERV3	LTR	1173	1111	901	980	766	692	666	777
LTR7C	ERV3	LTR	228	267	184	205	155	163	196	174
LTR7Y	ERV3	LTR	1894	1648	1362	1547	1196	1106	1383	1475
MER21A	ERV3	LTR	29	26	22	19	20	15	16	28
MER21B	ERV3	LTR	19	22	12	18	13	14	18	19
MER21C	ERV3	LTR	16	12	8	10	7	7	5	3
MLT1A0	ERV3	LTR	27	40	18	16	13	14	13	24
MLT1B	ERV3	LTR	107	91	65	81	66	60	42	61
MLT1C	ERV3	LTR	48	57	39	42	33	25	33	49
MLT1C1	ERV3	LTR	25	31	14	15	10	12	18	13
MLT1D	ERV3	LTR	37	35	27	26	27	17	22	28
MLT2A1	ERV3	LTR	530	482	259	331	312	306	277	345
MLT2A2	ERV3	LTR	817	774	429	482	479	451	393	517
MLT2B2	ERV3	LTR	23	20	12	16	9	8	6	10
MSTA	ERV3	LTR	609	632	409	475	362	351	317	425
MSTA1	ERV3	LTR	25	28	10	22	15	16	11	14
MSTA2	ERV3	LTR	44	43	43	33	28	21	21	21
MSTB	ERV3	LTR	188	213	163	158	115	113	118	149
MSTB1	ERV3	LTR	95	105	65	77	78	47	61	68
MSTC	ERV3	LTR	41	51	35	38	28	18	24	29
MSTD	ERV3	LTR	27	27	13	14	12	9	11	10
MST_I	ERV3	LTR	629	562	354	427	311	401	276	405
THE1A	ERV3	LTR	1047	912	692	735	605	648	486	674
THE1B	ERV3	LTR	1173	1196	778	858	691	616	579	700
THE1C	ERV3	LTR	638	632	386	395	368	333	302	402
THE1D	ERV3	LTR	517	558	334	344	262	242	194	286
L2	CR1	LINE	15	16	14	15	18	14	10	23
7SK	Pseudogene	Pseudo gene	26	17	19	23	25	22	28	19
7SL	Pseudogene	Pseudo gene	10023	9262	8440	8478	9026	8980	12982	14256
CYCLO	Pseudogene	Pseudo gene	140	134	77	110	107	110	95	128
L23	Pseudogene	Pseudo gene	97	103	95	84	101	77	78	80
L23A	Pseudogene	Pseudo gene	78	53	44	68	46	57	44	65
L28	Pseudogene	Pseudo gene	40	37	40	54	43	49	47	71
L31	Pseudogene	Pseudo gene	63	32	36	44	42	52	40	50
L37	Pseudogene	Pseudo gene	19	29	14	19	24	20	15	23
L7	Pseudogene	Pseudo gene	59	51	31	41	43	37	25	26
L7A	Pseudogene	Pseudo gene	164	195	169	178	151	163	162	152
RICKSHA	MuDR	DNA	15	15	12	16	6	7	10	9
HARLEQUINLTR	LTRRetro transpos	LTR	1355	1099	892	1101	1003	1070	1210	1204

	on									
HERV-K14CI	LTRRetro transpos on	LTR	79	81	57	64	57	38	36	44
HERV-K14I	LTRRetro transpos on	LTR	215	214	171	167	184	162	152	136
HUERS-P3	LTRRetro transpos on	LTR	48	53	31	39	34	22	18	26
LOR1	LTRRetro transpos on	LTR	11	11	12	13	5	8	11	4
MER4I	LTRRetro transpos on	LTR	65	62	43	42	45	60	43	61
MER51I	LTRRetro transpos on	LTR	26	25	12	12	14	11	12	15
MER52B	LTRRetro transpos on	LTR	108	110	75	102	85	101	100	98
MER61D	LTRRetro transpos on	LTR	24	14	8	11	6	12	13	10
MER61E	LTRRetro transpos on	LTR	16	25	15	18	18	16	15	11
MER61I	LTRRetro transpos on	LTR	139	101	69	77	55	66	54	61
PTR5	LTRRetro transpos on	LTR	586	627	638	673	631	607	1056	1089
THE1_I	LTRRetro transpos on	LTR	2919	2823	1776	1923	1561	1615	1492	1817
U1	snRNA	snRNA	33	25	21	16	22	13	15	28
U2	snRNA	snRNA	25	42	24	28	23	28	31	18
U6	snRNA	snRNA	99	68	59	84	71	80	61	59
5S	rRNA	rRNA	29	50	218	54	32	54	65	111
LSU-rRNA_Cel	rRNA	rRNA	1593	1381	802	2089	66	1412	164	319
LSU-rRNA_Dme	rRNA	rRNA	1577	1403	817	2063	46	1440	186	336
LSU-rRNA_Hsa	rRNA	rRNA	31177	27713	17765	42565	1290	28059	6417	10913
SSU-rRNA_Cel	rRNA	rRNA	1232	1173	733	1528	81	969	157	283
SSU-rRNA_Dme	rRNA	rRNA	1658	1498	947	2028	101	1273	217	404
SSU-rRNA_Hsa	rRNA	rRNA	13288	11085	7193	16585	867	10018	2065	3239
R66	R66	R66	106	67	42	61	35	39	36	45
MSR1	MSAT	Satellit e	17	34	23	22	20	8	27	29
SVA2	MSAT	Satellit e	43	40	43	33	27	30	39	54
HARLEQUIN	ERV1	LTR	739	696	516	570	500	551	479	575
HERV-Fc1	ERV1	LTR	323	370	379	387	274	287	356	318
HERV-Fc2	ERV1	LTR	175	225	224	244	213	160	203	193
HERV17	ERV1	LTR	1482	1346	1071	1193	981	965	895	1051
HERV19I	ERV1	LTR	49	51	38	47	32	33	26	34
HERV1_I	ERV1	LTR	77	70	53	84	62	54	58	58
HERV1_LTRb	ERV1	LTR	13	12	7	9	10	4	10	9
HERV3	ERV1	LTR	61	67	37	57	54	43	58	53
HERV35I	ERV1	LTR	23	18	12	18	9	17	7	5

HERV4_I	ERV1	LTR	32	42	32	17	25	23	19	18
HERV9	ERV1	LTR	847	992	688	722	545	499	498	535
HERVE	ERV1	LTR	1139	974	1042	970	861	795	846	922
HERVE_a	ERV1	LTR	1105	987	903	939	797	770	813	917
HERVH	ERV1	LTR	10971	10303	9135	9770	9366	8767	9701	9994
HERVI	ERV1	LTR	72	57	40	48	45	49	53	77
HERVIP10F	ERV1	LTR	385	366	314	306	293	254	257	300
HERVIP10FH	ERV1	LTR	1440	1239	1166	1505	820	1139	2115	2410
HERVS71	ERV1	LTR	276	307	303	330	250	234	294	275
HUERS-P1	ERV1	LTR	91	102	56	67	44	57	53	58
LOR1I	ERV1	LTR	36	24	14	19	13	14	13	19
LOR1a_LTR	ERV1	LTR	7	15	13	11	14	14	18	23
LTR1	ERV1	LTR	60	69	50	45	46	51	44	63
LTR10A	ERV1	LTR	126	137	116	134	104	112	128	145
LTR10B	ERV1	LTR	25	22	16	23	20	17	16	34
LTR10C	ERV1	LTR	122	141	95	79	72	58	66	91
LTR10D	ERV1	LTR	40	45	41	38	37	32	27	24
LTR10E	ERV1	LTR	27	34	23	16	15	17	12	19
LTR10F	ERV1	LTR	635	547	437	548	701	674	645	691
LTR10G	ERV1	LTR	58	69	57	52	64	65	57	57
LTR12	ERV1	LTR	1458	1243	1060	1164	858	948	858	915
LTR12B	ERV1	LTR	1295	1004	923	980	731	853	757	754
LTR12C	ERV1	LTR	16797	15601	14303	15832	13011	13272	18290	19132
LTR12D	ERV1	LTR	2883	2418	2134	2387	1862	1902	2808	2999
LTR12E	ERV1	LTR	8811	8750	7985	8903	7934	8090	13043	13068
LTR12F	ERV1	LTR	978	763	698	762	596	667	620	639
LTR15	ERV1	LTR	14	19	4	13	3	6	5	7
LTR17	ERV1	LTR	1862	1756	1346	1668	1581	1595	1678	1837
LTR1A2	ERV1	LTR	311	322	225	284	239	287	277	300
LTR1B	ERV1	LTR	91	77	49	64	66	59	61	67
LTR1B0	ERV1	LTR	56	58	48	78	52	55	47	69
LTR1B1	ERV1	LTR	85	80	50	75	70	66	62	73
LTR1C	ERV1	LTR	29	35	28	19	23	19	31	21
LTR1C1	ERV1	LTR	43	32	21	36	36	28	41	34
LTR1C2	ERV1	LTR	19	13	9	16	13	10	11	14
LTR1C3	ERV1	LTR	19	14	10	22	14	14	23	17
LTR1D	ERV1	LTR	72	53	31	41	29	42	42	51
LTR1D1	ERV1	LTR	60	39	37	33	33	33	36	39
LTR1E	ERV1	LTR	53	49	53	62	51	46	48	48
LTR1F	ERV1	LTR	49	47	33	47	44	38	35	50
LTR1F1	ERV1	LTR	35	24	17	24	12	16	18	25
LTR1F2	ERV1	LTR	53	44	36	62	44	41	43	55
LTR25-int	ERV1	LTR	23	16	7	10	8	1	7	11
LTR26	ERV1	LTR	20	12	15	12	8	5	9	6
LTR26B	ERV1	LTR	17	13	20	16	14	15	19	14
LTR27	ERV1	LTR	18	24	17	20	14	12	14	15
LTR2752	ERV1	LTR	56	55	35	51	38	47	66	58
LTR28	ERV1	LTR	39	30	22	22	23	17	27	26
LTR28B	ERV1	LTR	24	23	18	18	13	14	19	16
LTR28C	ERV1	LTR	27	41	19	22	30	21	19	29

LTR2B	ERV1	LTR	353	237	183	212	182	211	221	251
LTR2C	ERV1	LTR	50	58	41	52	46	38	38	46
LTR30	ERV1	LTR	48	39	27	42	26	20	24	27
LTR35A	ERV1	LTR	3	15	7	11	12	13	13	9
LTR39	ERV1	LTR	17	13	10	7	3	4	8	7
LTR43	ERV1	LTR	22	16	18	18	22	16	11	12
LTR45B	ERV1	LTR	18	18	7	17	7	11	8	6
LTR46	ERV1	LTR	23	12	9	11	16	12	15	15
LTR49	ERV1	LTR	25	33	12	15	20	15	14	21
LTR6A	ERV1	LTR	69	59	43	48	34	37	31	26
LTR6B	ERV1	LTR	127	118	108	100	78	53	75	71
LTR71A	ERV1	LTR	44	29	30	47	32	33	26	34
LTR71B	ERV1	LTR	63	59	58	40	40	41	39	35
LTR76	ERV1	LTR	15	22	13	13	15	13	14	11
LTR8	ERV1	LTR	261	238	199	213	171	155	158	176
LTR8A	ERV1	LTR	47	42	28	34	28	14	13	29
LTR8B	ERV1	LTR	34	27	20	14	24	11	23	28
LTR9	ERV1	LTR	145	108	100	97	97	98	83	96
LTR9A1	ERV1	LTR	54	45	37	41	37	37	36	32
LTR9B	ERV1	LTR	182	183	132	159	126	118	126	140
LTR9D	ERV1	LTR	37	35	28	28	27	19	15	30
MER31A	ERV1	LTR	16	19	13	20	11	20	9	9
MER39	ERV1	LTR	25	18	6	7	14	11	10	17
MER39B	ERV1	LTR	40	44	21	30	18	22	24	20
MER41A	ERV1	LTR	156	147	125	128	108	104	95	112
MER41B	ERV1	LTR	62	51	41	46	33	38	27	26
MER41C	ERV1	LTR	18	21	12	14	13	10	9	7
MER41D	ERV1	LTR	24	11	15	14	17	7	13	9
MER41E	ERV1	LTR	19	11	9	8	12	14	10	9
MER48	ERV1	LTR	82	93	80	109	101	98	90	106
MER49	ERV1	LTR	34	32	22	23	18	13	14	22
MER4A	ERV1	LTR	41	41	22	28	28	19	18	20
MER4A1	ERV1	LTR	264	234	186	221	144	138	112	148
MER4A1_LTR	ERV1	LTR	129	119	100	108	70	70	74	72
MER4B	ERV1	LTR	34	20	12	17	8	11	6	11
MER4C	ERV1	LTR	36	31	21	19	10	14	18	20
MER4D	ERV1	LTR	26	38	19	24	22	14	13	9
MER4D1	ERV1	LTR	76	65	39	49	39	28	22	30
MER4D_LTR	ERV1	LTR	38	38	36	40	21	25	13	22
MER4E	ERV1	LTR	134	102	61	81	62	62	37	57
MER4E1	ERV1	LTR	138	91	61	84	51	69	41	68
MER50	ERV1	LTR	55	45	37	49	44	36	27	43
MER50B	ERV1	LTR	24	25	25	30	22	20	18	27
MER50C	ERV1	LTR	16	18	13	16	18	8	11	25
MER50I	ERV1	LTR	37	47	26	33	32	24	21	29
MER51A	ERV1	LTR	77	64	39	56	39	49	40	50
MER51B	ERV1	LTR	87	71	48	47	36	49	43	41
MER51C	ERV1	LTR	22	20	21	19	21	14	16	13
MER51E	ERV1	LTR	16	15	10	7	9	14	6	15
MER52A	ERV1	LTR	124	102	80	82	72	88	89	111

MER52AI	ERV1	LTR	35	48	27	19	20	16	15	21
MER52C	ERV1	LTR	67	80	55	60	53	48	61	80
MER52D	ERV1	LTR	86	100	58	84	72	82	106	118
MER57A1	ERV1	LTR	88	88	60	79	66	63	48	61
MER57B1	ERV1	LTR	55	44	44	54	41	44	44	38
MER57B2	ERV1	LTR	16	12	13	14	11	20	11	14
MER61C	ERV1	LTR	13	14	8	13	8	4	6	10
MER66C	ERV1	LTR	37	42	22	23	23	16	22	26
MER66_I	ERV1	LTR	14	14	7	6	10	9	5	12
MER83	ERV1	LTR	20	29	16	18	22	11	12	21
MER83C	ERV1	LTR	16	10	8	10	14	14	9	13
PABL_A	ERV1	LTR	44	52	45	46	33	31	31	35
PABL_AI	ERV1	LTR	45	26	22	35	22	25	25	35
PABL_B	ERV1	LTR	19	20	14	19	15	11	15	11
PRIMA4_LTR	ERV1	LTR	34	26	20	27	18	12	13	16
MER122	MER122	MER122	24	37	33	38	31	29	40	32
MIR	SINE	SINE	24	16	10	9	8	7	3	5
SVA	SINE	SINE	64185	64538	63583	66819	59089	60622	104990	104893
ACRO1	SAT	Satellite	3028	4172	3750	3847	2181	2850	3560	2767
ALR	SAT	Satellite	4498	5036	5060	5239	1752	1341	1265	1511
ALR1	SAT	Satellite	8731	8045	8053	7146	3224	2429	2119	2786
ALR2	SAT	Satellite	49	71	54	39	25	17	18	22
ALR_	SAT	Satellite	3973	5132	4210	4188	2029	1494	1002	1179
ALRa_	SAT	Satellite	17	31	19	17	8	8	6	7
ALRb	SAT	Satellite	5316	6230	6021	5915	2518	1978	1555	1645
BSR	SAT	Satellite	582	876	842	838	540	317	346	422
CER	SAT	Satellite	104	168	100	123	86	66	55	74
D20S16	SAT	Satellite	30	52	35	32	23	40	36	30
GSAT	SAT	Satellite	109	139	151	146	103	96	154	127
GSATII	SAT	Satellite	183	189	227	193	125	126	250	199
GSATX	SAT	Satellite	28	45	40	43	25	29	37	43
HSAT5	SAT	Satellite	6	9	10	15	12	6	8	3
HSATI	SAT	Satellite	364	77	57	47	9	12	14	15
LSAU	SAT	Satellite	476	690	884	957	542	427	788	730
REP522	Satellite	Satellite	46	51	36	52	43	31	67	66
TAR1	Satellite	Satellite	1069	803	778	736	914	694	1749	1373
Hg18	Hg18	Hg18	6170830	6378017	6451329	6248898	6124398	5500468	6987639	6865064
