# Supplementary material for: The landscape of DNA repeat elements in human heart failure
Source: Genome Biol. 2012 Oct 3;13(10):R90. doi: 10.1186/gb-2012-13-10-r90 (PMC3491418; doi:10.1186/gb-2012-13-10-r90)
Supplement: Additional file 10 — List of all ALR, ALR_ and ALRb elements and coordinates in the human genome according to Hg18. [file gb-2012-13-10-r90-S10.pdf]

Id	Repeat	Chr	Strand	Start	End	Gene Symbol (5prime 5000bp upstream)
39	ALR	chr3	-	90548062	90548093	
40	ALR	chr3	-	90489240	90489271	
28	ALR	chr4	-	67947143	67947579	
27	ALR	chr7	+	61613934	61614039	
32	ALR	chr7	+	61612596	61612674	
20	ALR	chr7	+	61607485	61607597	
14	ALR	chr7	+	61605783	61643936	
22	ALR	chr7	+	61613594	61613706	
30	ALR	chr7	+	61611236	61611331	
16	ALR	chr7	+	61606463	61612409	
19	ALR	chr7	+	61605274	61605386	
29	ALR	chr7	+	61611916	61612011	
31	ALR	chr7	+	61611407	61611671	
5	ALR	chr7	+	61604594	61617838	
42	ALR	chr9	+	66551599	66551628	
35	ALR	chr19	+	32429339	32429382	
11	ALR	chr19	+	32430358	32430528	
3	ALR	chr19	+	32423912	32424082	
7	ALR	chr19	+	32429000	32429508	
9	ALR	chr19	+	32425940	32426110	
2	ALR	chr19	+	32424252	32425771	
15	ALR	chr19	+	32428320	32428470	
38	ALR	chr19	-	32596387	32596421	
8	ALR	chr19	+	32432054	32432927	
4	ALR	chr19	+	32430698	32431884	
18	ALR	chr19	+	32425269	32426790	
37	ALR	chr19	-	32590023	32590062	
10	ALR	chr19	+	32431038	32431208	
1	ALR	chr1	+	121185594	121185932	
0	ALR	chr1	+	121186613	121186783	
34	ALR	chr11	-	50637083	50713081	
41	ALR	chr11	+	48786990	48787021	
36	ALR	chr19_random	-	51538	51587	
25	ALR	chr10	-	41857748	41861256	
6	ALR	chr10	+	41720099	41720269	
21	ALR	chr10	-	41849262	41849374	
26	ALR	chr10	-	41856051	41858706	
17	ALR	chr10	+	41718575	41718744	
12	ALR	chr10	+	41719253	41719423	
24	ALR	chr10	+	41722303	41722413	
13	ALR	chr10	-	41858369	41864994	
23	ALR	chr10	+	41718914	41719026	
33	ALR	chr16	-	33899311	33899626	
274	ALRb	chr7	+	61604680	61605868	
330	ALRb	chr7	+	57961948	57982951	
307	ALRb	chr7	+	61604594	61604679	

295	ALRb	chr7	+	61613680	61613806	
287	ALRb	chr7	+	61607231	61607570	
336	ALRb	chr7	+	61609948	61609978	
277	ALRb	chr7	+	61605869	61607910	
329	ALRb	chr7	+	61605913	61613679	
275	ALRb	chr7	+	61606549	61606889	
284	ALRb	chr7	+	61607571	61610286	
301	ALRb	chr7	+	61605026	61605359	
322	ALRb	chr20	+	26212769	26213061	
303	ALRb	chr14	-	18105583	18105686	
296	ALRb	chr8	+	43892926	43893579	
315	ALRb	chr19	+	32428320	32429934	
276	ALRb	chr19	+	32423998	32425004	
320	ALRb	chr19	+	32430784	32430865	
309	ALRb	chr19	+	32424252	32428065	
278	ALRb	chr19	+	32428066	32437643	
266	ALRb	chr19	+	32429764	32430778	
331	ALRb	chr19	+	32430018	32430056	
271	ALRb	chr1	+	121186020	121186359	
321	ALRb	chr1	+	121186699	121186768	
318	ALRb	chr1	+	121186630	121186698	
302	ALRb	chr11	+	48816882	48819379	
337	ALRb	chr11	+	48819495	48819517	
298	ALRb	chr11	+	54766184	54766474	
292	ALRb	chr11	+	54768889	54769203	
323	ALRb	chr11	-	48892530	48892592	
316	ALRb	chr11	+	48785761	48786007	
290	ALRb	chr11	+	50657350	50657690	
297	ALRb	chr21	-	13290008	13290297	
312	ALRb	chr18	+	15371978	15372064	
313	ALRb	chr3	-	90436342	90436944	
334	ALRb	chr12	+	34742862	34743063	
304	ALRb	chr12	+	36304209	36304317	
310	ALRb	chr12	+	34730924	34731009	
314	ALRb	chr12	+	36281989	36304007	
319	ALRb	chrX	+	61759330	61759575	
326	ALRb	chrX	+	61675739	61675793	
300	ALRb	chr2	+	132713494	132714300	ANKRD30BL
286	ALRb	chr9	+	66540569	66540729	
288	ALRb	chr9	+	69286462	69286788	
285	ALRb	chr9	+	69280316	69280476	
294	ALRb	chr19_random	-	27687	90845	
325	ALRb	chr19_random	-	90533	90588	
328	ALRb	chr10	+	41719386	41720862	
282	ALRb	chr10	-	41849289	41849621	
267	ALRb	chr10	+	41718661	41719338	
327	ALRb	chr10	+	41722095	41724587	
281	ALRb	chr10	-	41848268	41849287	
269	ALRb	chr10	+	41719339	41726616	
324	ALRb	chr10	-	41855066	41855123	

333	ALRb	chr10	-	41847562	41847590	
272	ALRb	chr10	-	41853361	41855050	
280	ALRb	chr10	-	41850649	41858444	
283	ALRb	chr10	-	41847251	41861502	
291	ALRb	chr10	+	41718710	41718999	
332	ALRb	chr10	+	41722394	41726341	
273	ALRb	chr10	-	41850309	41850648	
305	ALRb	chr10	+	41718575	41718660	
268	ALRb	chr10	+	41720523	41722388	
279	ALRb	chr10	+	41719000	41727301	
299	ALRb	chr10	-	41861861	41862144	
317	ALRb	chr10	-	41854726	41854804	
265	ALRb	chr10	+	41720185	41720524	
270	ALRb	chr10	+	41718321	41720184	
311	ALRb	chr10	+	41719879	41724720	
308	ALRb	chr5	-	49442753	49443046	
306	ALRb	chr5	-	49453819	49456328	
289	ALRb	chr5	-	46388402	46388541	
335	ALRb	chr5	-	49467281	49467329	
293	ALRb	chr5	-	49456033	49467755	
67	ALR_	chr7	+	61088208	61088378	
207	ALR_	chr7	-	61668891	61670727	
262	ALR_	chr7	+	61631205	61631224	
167	ALR_	chr7	-	61670728	61670883	
99	ALR_	chr7	-	58003882	58014811	
77	ALR_	chr7	+	61087354	61087524	
190	ALR_	chr7	+	61624219	61624387	
203	ALR_	chr7	+	61617470	61617584	
259	ALR_	chr7	-	61647392	61647413	
253	ALR_	chr7	-	61668684	61668705	
108	ALR_	chr7	-	61671510	61671850	
114	ALR_	chr7	-	61647243	61669023	
146	ALR_	chr7	+	61091784	61091946	
199	ALR_	chr7	-	58003920	58004052	
58	ALR_	chr7	+	61084118	61084799	
115	ALR_	chr7	-	58010710	58010880	
137	ALR_	chr7	+	61629501	61631033	
66	ALR_	chr7	+	61637869	61638209	
216	ALR_	chr7	+	61629600	61629668	
247	ALR_	chr7	-	58014474	58014500	
74	ALR_	chr20	+	26237177	26254987	
130	ALR_	chr20	+	26259429	26259599	
182	ALR_	chr20	+	26210710	26211038	
241	ALR_	chr20	+	26219900	26219927	
206	ALR_	chr20	+	26213272	26213386	
213	ALR_	chr20	+	26237084	26250153	
97	ALR_	chr20	+	26249538	26249708	
188	ALR_	chr20	+	26253633	26255123	
129	ALR_	chr20	+	26213217	26253794	
106	ALR_	chr20	+	26246975	26247145	

164	ALR_	chr20	+	26236159	26237176	
140	ALR_	chr20	+	26219757	26236497	
197	ALR_	chr14	-	18077982	18082542	
252	ALR_	chr14	-	18111552	18111576	
194	ALR_	chr14	-	18106265	18111334	
156	ALR_	chr14	-	18126266	18126436	
95	ALR_	chr14	-	18105076	18111722	
83	ALR_	chr14	-	18084080	18091588	
248	ALR_	chr14	-	18111040	18111064	
239	ALR_	chr14	-	18091346	18091542	
139	ALR_	chr14	-	18082199	18105246	
135	ALR_	chr14	-	18113262	18113431	
119	ALR_	chr14	-	18077941	18092612	
103	ALR_	chr14	-	18078623	18084250	
179	ALR_	chr14	-	18075761	18076238	
46	ALR_	chr14	-	18080498	18111380	
171	ALR_	chr14	-	18091760	18106093	
222	ALR_	chr14	-	18106316	18106361	
128	ALR_	chr8	+	43889685	43889850	
105	ALR_	chr8	+	43906716	43907057	
131	ALR_	chr19	+	24417371	24418906	
122	ALR_	chr19	+	32460916	32462449	
48	ALR_	chr19	+	32454190	32462619	
162	ALR_	chr19	+	24395402	24395914	
144	ALR_	chr19	+	32437815	32437984	
264	ALR_	chr19	+	32462279	32462298	
133	ALR_	chr19	+	24400171	24401706	
110	ALR_	chr19	+	32462450	32470720	
159	ALR_	chr19	+	32459235	32461086	
161	ALR_	chr19	+	24398298	24398810	
185	ALR_	chr19	+	24415670	24417540	
94	ALR_	chr19	-	32578868	32579037	
236	ALR_	chr19	+	24418565	24418775	
160	ALR_	chr19	+	24412775	24413116	
184	ALR_	chr19	+	32454221	32454533	
211	ALR_	chr19	+	24400251	24400340	
173	ALR_	chr19	+	24401365	24414645	
229	ALR_	chr19	+	32470041	32470080	
132	ALR_	chr19	+	24414476	24416011	
242	ALR_	chr19	+	32459543	32459570	
123	ALR_	chr6	-	61959725	61960062	
200	ALR_	chr6	-	61974235	61974349	
260	ALR_	chr6	-	61962600	61962619	
202	ALR_	chr6	-	61972184	61972306	
54	ALR_	chr6	-	61962449	61972352	
90	ALR_	chr11	+	48785837	48819892	
71	ALR_	chr11	-	54779356	54782252	TRIM48
227	ALR_	chr11	+	48786315	48786348	
244	ALR_	chr11	-	54778130	54778328	
136	ALR_	chr11	-	54778158	54779682	

88	ALR_	chr11	-	48891818	48892187	
72	ALR_	chr11	-	48893383	48893553	
240	ALR_	chr11	-	54782082	54782111	TRIM48
120	ALR_	chr11	-	48774145	48774826	
57	ALR_	chr11	-	48904961	48905131	
192	ALR_	chr11	+	48786053	48786178	
59	ALR_	chr11	+	48786179	48821996	
92	ALR_	chr11	+	48834143	48834309	
178	ALR_	chr11	+	48819722	48823708	
151	ALR_	chr11	-	54770140	54770310	
89	ALR_	chr11	-	48914933	48915103	
148	ALR_	chr11	+	48789994	48790163	
104	ALR_	chr11	-	54773726	54773896	
155	ALR_	chr11	+	48819210	48819379	
189	ALR_	chr11	-	48920405	48920527	
225	ALR_	chr11	-	48913177	48913563	
91	ALR_	chr11	+	48824905	48825246	
205	ALR_	chr11	-	48913393	48913495	
154	ALR_	chr11	+	48852989	48853138	
214	ALR_	chr11	-	54779398	54779479	
257	ALR_	chr11	+	48852797	48852817	
96	ALR_	chr11	-	54774740	54775080	
85	ALR_	chr11	+	48836362	48836703	
258	ALR_	chr11	+	48821826	48821848	
153	ALR_	chr11	+	48849746	48849916	
98	ALR_	chr11	+	48850942	48851112	
56	ALR_	chr11	-	48902903	48903244	
86	ALR_	chr11	+	48849233	48849403	
62	ALR_	chr11	-	48909974	48910315	
81	ALR_	chr21	-	13287448	13287618	
201	ALR_	chr21	-	13260062	13260368	
118	ALR_	chr21	-	13260027	13273084	
102	ALR_	chr21	-	13290008	13290347	
224	ALR_	chr21	-	13272914	13290472	
245	ALR_	chr16	+	35141344	35141712	
177	ALR_	chr16	+	35103896	35104060	
196	ALR_	chr16	+	35141570	35141684	
176	ALR_	chr16	+	35108672	35108836	
238	ALR_	chr3	-	90499730	90499757	
149	ALR_	chr3	-	90571139	90573856	
124	ALR_	chr3	-	90573008	90573178	
210	ALR_	chr3	-	90499627	90542374	
230	ALR_	chr3	-	90579814	90579844	
168	ALR_	chr3	-	90499587	90538314	
101	ALR_	chr3	-	90576071	90576410	
255	ALR_	chr3	-	90542034	90542054	
43	ALR_	chr3	-	90538144	90571306	
117	ALR_	chr3	-	90532983	90533322	
175	ALR_	chr3	-	90573689	90580154	
187	ALR_	chr3	-	90549178	90549342	

223	ALR_	chr3	-	90538657	90573810	
55	ALR_	chr3	-	90531107	90538998	
49	ALR_	chr15	-	18265018	18265700	
226	ALR_	chr15	-	18265189	18265358	
147	ALR_	chr12	+	34743370	34743530	
84	ALR_	chr12	+	34729179	34729683	
233	ALR_	chr12	+	36301789	36301825	
165	ALR_	chr12	-	36319440	36319608	
68	ALR_	chr12	+	36282954	36301959	
166	ALR_	chr12	+	36297256	36297420	
215	ALR_	chr12	+	36301845	36301923	
232	ALR_	chr12	+	36283430	36283634	
157	ALR_	chr12	-	36316546	36316711	
237	ALR_	chrX	-	61660087	61660123	
152	ALR_	chrX	+	61666159	61666643	
217	ALR_	chrX	+	61743881	61746611	
204	ALR_	chrX	-	61660457	61660739	
53	ALR_	chrX	-	61659953	61660966	
234	ALR_	chrX	+	61728451	61728485	
142	ALR_	chrX	-	58575808	58576146	
107	ALR_	chrX	-	61663679	61664017	
44	ALR_	chrX	+	61734152	61741834	
126	ALR_	chrX	+	61678865	61734322	
75	ALR_	chrX	+	61746442	61750537	
250	ALR_	chrX	+	61772119	61772142	
47	ALR_	chrX	+	61739277	61739789	
52	ALR_	chrX	+	61731759	61731929	
60	ALR_	chrX	-	61659444	61659781	
172	ALR_	chrX	+	58568811	58568981	
209	ALR_	chrX	+	61771975	61772280	
256	ALR_	chrX	+	61679171	61679191	
76	ALR_	chrX	+	61728317	61744051	
87	ALR_	chr2	+	132714985	132715325	ANKRD30BL
174	ALR_	chr2	+	132692441	132713790	ANKRD30BL
263	ALR_	chr2	+	132713962	132713981	ANKRD30BL
249	ALR_	chr2	+	132692758	132692782	ANKRD30BL
235	ALR_	chr2	+	132713723	132713755	ANKRD30BL
218	ALR_	chr2	+	132714003	132714063	ANKRD30BL
143	ALR_	chr2	+	132690507	132690901	ANKRD30BL
113	ALR_	chr2	+	132703602	132703943	ANKRD30BL
63	ALR_	chr9	+	69270265	69288153	
198	ALR_	chr9	+	69287526	69287641	
158	ALR_	chr9	+	69238202	69238372	
212	ALR_	chr9	+	69270511	69270606	
186	ALR_	chr9	+	66528010	66551654	
65	ALR_	chr9	+	66557123	66562236	
221	ALR_	chr9	+	66562237	66562295	
251	ALR_	chr9	+	66528116	66528139	
261	ALR_	chr9	+	69287812	69287831	
64	ALR_	chr9	+	66527628	66548415	

228	ALR_	chr9	+	69270647	69270681	
220	ALR_	chr9	+	66548074	66561834	
109	ALR_	chr9	+	66555415	66555755	
254	ALR_	chr9	+	66557101	66557122	
145	ALR_	chr9	+	67008080	67008234	
73	ALR_	chr9	+	66551313	66555242	
170	ALR_	chr19_random	-	37714	39076	
50	ALR_	chr19_random	-	4305	85594	
134	ALR_	chr19_random	-	16545	16715	
70	ALR_	chr19_random	-	23593	36351	
51	ALR_	chr19_random	-	47235	47405	
193	ALR_	chr19_random	-	21545	85547	
69	ALR_	chr19_random	-	27516	37881	
112	ALR_	chr19_random	-	49795	71783	
191	ALR_	chr19_random	-	89340	89478	
180	ALR_	chr19_random	-	29052	29390	
93	ALR_	chr19_random	-	11120	21714	
169	ALR_	chr19_random	-	49112	49282	
125	ALR_	chr19_random	-	10607	10777	
45	ALR_	chr19_random	-	45871	89510	
150	ALR_	chr19_random	-	39928	40091	
82	ALR_	chr19_random	-	38222	38392	
231	ALR_	chr9_random	+	389768	389798	
163	ALR_	chr9_random	+	398402	398742	
127	ALR_	chr9_random	+	389638	410520	
219	ALR_	chr9_random	+	410520	410593	
61	ALR_	chr9_random	-	936890	937400	
121	ALR_	chr9_random	+	394008	394337	
79	ALR_	chr9_random	-	935534	935875	
246	ALR_	chr5	-	46392814	46392839	
181	ALR_	chr5	+	46414728	46415054	
183	ALR_	chr5	-	46379361	46379702	
138	ALR_	chr5	-	46368838	46369172	
208	ALR_	chr5	-	46372089	46372218	
111	ALR_	chr5	-	46375276	46375617	
80	ALR_	chr5	-	49465568	49466080	
78	ALR_	chr5	+	46404427	46415410	
243	ALR_	chr5	-	46377121	46377319	
116	ALR_	chr5	-	46372050	46392975	
141	ALR_	chr5	+	46427314	46427484	
100	ALR_	chr5	-	46376978	46380381	
195	ALR_	chr5	+	46404820	46405094	
