# Supplementary material for: The landscape of DNA repeat elements in human heart failure
Source: Genome Biol. 2012 Oct 3;13(10):R90. doi: 10.1186/gb-2012-13-10-r90 (PMC3491418; doi:10.1186/gb-2012-13-10-r90)
Supplement: Additional file 13 — Table S2 - list of LV sample details. [file gb-2012-13-10-r90-S13.docx]

**Supplementary Table 2.** List of LV sample details.

|  | **LV sample** | **age** | **details** | **medications** |
| --- | --- | --- | --- | --- |
| Control  (CTRL) | A | 47 | RTA | - |
|  | B | 50 | RTA | - |
|  | C | 41 | RTA | - |
|  | D | 52 | RTA | - |
|  | E | 58 | RTA | - |
|  | F | 47 | Hypoxic brain injury secondary to drowning | - |
|  | G | 53 | RTA | - |
|  | H | 54 | RTA | - |
| End-stage cardiomyopathy  (EsCM) | 1 | 42 | Idiopathic | Aspirin, perindopril, carvedilol, warfarin |
|  | 2 | 64 | Idiopathic | Frusemide, spironolactone, lisinopril, amiodarone, atorvastatin, warfarin |
|  | 3 | 61 | Ischemic | Aspirin, clopidogrel, lisinopril, simvastatin, bisoprolol, spironolactone |
|  | 4 | 60 | Ischemic | Clopidogrel, bisoprolol, ramipril, spironolactone, simvastatin |
|  | 5 | 53 | Idiopathic | Frusemide, spironolactone, lisinopril, atorvastatin, warfarin |
|  | 6 | 64 | Ischemic | Clopidogrel, ramipril, spironolactone |
|  | 7 | 58 | Ischemic | Clopidogrel, ramipril, carvedilol, spironolactone, frusemide |
|  | 8 | 49 | Idiopathic | Candesartan, warfarin |
|  | 9 | 42 | Idiopathic | Carvedilol, frusemide, lisinopril, spironolactone warfarin |
|  | 10 | 51 | Ischemic | Spironolactone, aspirin, amiodarone, frusemide |
|  | 11 | 41 | Idiopathic | Frusemide, spironolactone |
|  | 12 | 55 | Ischemic | Warfarin, aspirin, bumetanide |
|  | 13 | 49 | Idiopathic | Ramipril, spironolactone, frusemide |
|  | 14 | 19 | Idiopathic | Candesartan, spironolactone, bumetanide |
|  | 15 | 61 | Idiopathic | Candesartan, warfarin, spironolactone, frusemide, amiodarone |
|  | 16 | 48 | Idiopathic | Candesartan, spironolactone, frusemide, warfarin |
| RTA: road traffic accident; all LV were from Caucasian males.  All idiopathic cardiomyopathic patients had no history of coronary artery disease. | | | | |
