# Supplementary material for: The landscape of DNA repeat elements in human heart failure
Source: Genome Biol. 2012 Oct 3;13(10):R90. doi: 10.1186/gb-2012-13-10-r90 (PMC3491418; doi:10.1186/gb-2012-13-10-r90)
Supplement: Additional file 15 — Table S3 - genes related to SAT elements. [file gb-2012-13-10-r90-S15.docx]

# Supplementary Table 3. Genes related to SAT elements.

| **HGNC gene symbol** | **UCSC gene description** |
| --- | --- |
| *TRIM48* | Tripartite motif-containing protein 48 (RING finger protein 101) |
| *ANKRD30BL* | Ankyrin repeat domain 30B, non-protein coding RNA |
