# Supplementary material for: The landscape of DNA repeat elements in human heart failure
Source: Genome Biol. 2012 Oct 3;13(10):R90. doi: 10.1186/gb-2012-13-10-r90 (PMC3491418; doi:10.1186/gb-2012-13-10-r90)
Supplement: Additional file 16 — Table S4 - primers used for quantification PCR for SAT repeat elements. [file gb-2012-13-10-r90-S16.docx]

# Supplementary Table 4. Primers used for quantification PCR for SAT repeat elements.

| **Repeat** | **Forward primer** | **Reverse primer** |
| --- | --- | --- |
| ALR | ttctcagtaacttccttgtgttgtg | ggaagatatttcctattctaccattga |
| ALR_ | gaatctgcaagtggatatttgga | tcaaaactgctctatcaaaaggaa |
| ALRb | ttgtggaatttgcaagtgga | tgctctgtctaaaggaaggttca |
